# *Thyrostroma parviniae* sp. nov., causing bud necrosis and branch dieback in fig trees from Iran

**DOI:** 10.1371/journal.pone.0341992

**Published:** 2026-04-08

**Authors:** Hamed Negahban, Zeinab Bolboli, Moslem Jafari, Reza Mostowfizadeh-Ghalamfarsa

**Affiliations:** 1 Department of Plant Protection, School of Agriculture, Shiraz University, Shiraz, Iran; 2 Fig Research Station, Fars Agricultural and Natural Resources Research and Education Center, Agricultural Research, Education and Extension Organization (AREEO), Estahban, Iran; Universitat Jaume 1, SPAIN

## Abstract

Surveys were conducted in commercial fig orchards in Fars Province, Iran, from 2023 to 2024, focusing on symptomatic trees showing bud necrosis, branch dieback, and wood discoloration. Isolates with characteristic features of the genus *Thyrostroma* were isolated from diseased samples in Estahban, Ij, Qalat, and Shiraz Counties. Phylogenetic analyses using multigene sequence data from the *tef1*, *tub2*, ITS, and LSU loci, combined with morphological characteristics, revealed a new species of *Thyrostroma*, described here as *Thyrostroma parviniae* sp. nov. All *T. parviniae* sp. nov. isolates induced necrotic lesions on detached shoots, and principal component analysis showed a high degree of variation in aggressiveness. Pathogenicity was unequivocally established via Koch’s postulates, with artificial inoculations on fig saplings reproducing field symptoms on shoots, stems, and buds. Given its broad tissue tropism and ability to infect unwounded tissues in fig orchards, we hypothesize that *T. parviniae* sp. nov. may utilize airborne or latent infection mechanisms. This represents a key area for future investigation. The pathogen specifically targets primary buds, which are the most productive, indicating a significant potential for yield loss. Considering that figs are a widely traded commodity and plant material is transported internationally, the pathogen’s emergence poses a serious threat to global fig production. This threat warrants urgent research into molecular diagnostics, host range expansion risks, cultivar susceptibility, and climate-influenced epidemiology to guide effective fig disease management.

## Introduction

*Ficus carica* L. (*Moraceae*), the common fig, was domesticated approximately 11,000 years ago, making it one of the earliest cultivated fruit species [1]. Originating in Southwest Asia and the Middle East, this species exhibits remarkable adaptability to harsh environmental conditions, including poor soils and water scarcity, while providing substantial nutritional and health benefits [2–4]. Among its cultivated varieties, the Smyrna-type fig, comprising both fresh and dried forms, holds significant economic importance, particularly in Iran, where it is a major horticultural crop. *Ficus carica* ‘Sabz’ is a commercially important cultivar that predominates in Iran, representing 95% national fig production, while *F. carica* ‘Siah’ is widely cultivated for fresh consumption. It is the main cultivar used in arid and semi-arid plantations due to its strong ability to adapt to abiotic stresses such as drought. The ‘Sabz’ cultivar is well-known for producing the highest quantity and quality of dried fruits among Iranian fig genotypes [5]. Iran ranks among the world’s top producers and exporters with an annual output of 73,483.39 tons [6]. Within Iran, Fars Province is the leading region for dried fig production due to its ideal agroecological conditions and traditional rain-fed cultivation methods, which yield a high-quality, healthy product [5]. However, the productivity of this vital crop is threatened by various diseases, with dieback is particularly destructive.

Dieback disease, caused by various fungal pathogens, poses a significant threat to economically important trees, resulting in substantial economic losses and ecological impacts. The disease could negatively affect biodiversity, timber industries, and orchard productivity [7–9]. In edible fig trees, fungal dieback has been attributed to several pathogenic fungi, including, *Ceratocystis ficicola* Kajitani & Masuya in Italy [10], *Lasiodiplodia brasiliensis* M.S.B. Netto, M.W. Marques & A.J.L. Phillips in Malaysia [11], *L. theobromae* (Pat.) Griffon & Maubl. in Korea, Malaysia, Oman, Tunisia, and Turkey [11–15], *Neoscytalidium dimidiatum* (Penz.) Crous & Slippers in Australia, California, and Turkey [16–18], *Neofusicoccum parvum* (Pennycook & Samuels) Crous, Slippers & A.J.L. Phillips in Italy [19], and *Stilbocrea banihashemiana* Bolboli, Tavakolian & Mostowf. in Iran [20]. Notably, many of these fungal pathogens belong to the class *Dothideomycetes*, and the family *Botryosphaeriaceae*, and a comprehensive understanding of dieback requires consideration of other related pathogenic genera within this group, such as those in the family *Dothidotthiaceae*.

Members of the *Dothidotthiaceae* family are frequently reported as plant pathogens, saprobes, or endophytes, inhabiting leaves and woody tissues in terrestrial ecosystems [21,22]. Within this family, species of *Thyrostroma* are globally distributed pathogens known to cause canker and dieback on a phylogenetically diverse range of woody hosts [22–24]. To date, numerous *Thyrostroma* species have been described, including pathogens on economically and ecologically important hosts across diverse families such as *Cannabaceae*, *Cornaceae*, *Ephedraceae*, *Fabaceae*, *Moraceae*, *Solanaceae*, and *Ulmaceae* [22–26] (see S1 Table for a complete list of species and associated hosts). Notably, recent investigations have demonstrated the pathogenic ability of *T. cornicola* on wild almond trees (*Amygdalus scoparia* Spach) and an undescribed *Thyrostroma* sp. on grapevines (*Vitis vinifera* L.) [25,26]. Despite the established pathogenicity of *Thyrostroma* species on this wide range of hosts, their role in fig dieback had remained unexplored, with no previous reports of the genus on fig trees in the literature.

Recent surveys in the major fig-producing regions of Fars Province, Iran, identified a previously unidentified *Thyrostroma* species associated with twig dieback and bud necrosis. The disease specifically targets primary buds, which are the most productive, indicating a significant potential for yield loss. Considering that figs are a widely traded commodity and plant material such as cuttings is transported internationally, the emergence of this pathogen poses a potential threat to global fig production. This work addresses fundamental knowledge gaps by (i) characterizing *Thyrostroma* sp. isolates from diseased figs via integrated morphology-phylogeny studies, (ii) assessing the pathogenic potential of these isolates on detached shoots *in vitro*, (iii) quantifying the aggressiveness of studied isolates using various pathogenicity traits, (iv) fulfilling Koch’s postulates on one-year-old saplings under controlled conditions, and (v) evaluating leaf and fruit pathogenicity to assess crop-wide risks. By addressing these objectives, this study seeks to elucidate the etiology of fig dieback in a key production region, providing critical insights for disease management and biosecurity in fig cultivation systems.

## Materials and methods

### Sampling and fungal isolation

Surveys were conducted during the 2023–2024 growing season in selected fig production areas of Fars Province, Iran, including Estahban, Ij, Qalat, and Shiraz. Mature branches and one-year-old shoots exhibiting dieback symptoms were collected from infected fig trees. The symptomatic wood tissues were washed under running tap water, and cross-sectional pieces of discolored vascular tissues were prepared. Small fragments (25 mm^2^) were excised between necrotic and healthy tissues using a sterile scalpel. Following a 1-minute surface disinfection in 1.5% sodium hypochlorite solution (NaClO), samples were triple-rinsed in sterile distilled water, and dried on sterile absorbent paper under a laminar flow hood. The specimens were then placed on potato dextrose agar (PDA; 300 g/L boiled potato extract, 20 g/L glucose monohydrate, 15 g/L agar, and distilled water, supplemented with 100 mg/L ampicillin as bacterial growth inhibitor). The Petri dishes were incubated at 25 ± 3 °C for 7–10 days, allowing fungal colonies to develop sufficiently for examination. Emerging fungal colonies were purified through successive transfers to water agar (WA; 20 g/L agar, and distilled water) for single-hyphal-tip isolation. For long-term preservation, pure isolates were sub-cultured onto PDA plates containing sterilized filter paper pieces. Once the fungus had completely colonized the paper, they were transferred to sterile paper pockets and air-dried at room temperature. The dried, colonized filter papers were cut into small pieces, placed in 1.5 ml microtubes and stored at −20 °C [27].

### Morphological characterization

The morphological characteristics of fungal isolates were evaluated using established protocols [22]. Mycelial plugs (5 mm diameter) from 10-day-old cultures were transferred to PDA plates and incubated at 25 °C under a 12-hour light/dark photoperiod for 2–3 weeks. Colony morphology, pigmentation (surface and reverse) [28], and sporodochia development were assessed twice weekly. For microscopic analysis, fungal structures were mounted in lactic acid (60%) and examined using light microscopy (Carl Zeiss^®^, Axiophot, Germany). Conidial morphology and dimensions (length and width) were determined by measuring at least 30 randomly selected conidia, conidiophores, and conidiogenous cells per isolate. Radial growth rates were assessed by inoculating PDA plates (three replicates per isolate) with mycelial plugs from colony margins and incubating them in darkness at 5, 10, 15, 20, 25, 30, 35, and 40 °C for 10 days. Colony diameters were measured daily, and growth rates (mm/day) were calculated by linear regression of radial growth against time.

### DNA extraction, PCR amplification, and sequencing

Total genomic DNA was extracted from mycelium cultivated on PDA after a two-week incubation period, using the DNG-PLUS extraction kit (CinnaGen, Tehran, Iran), following the manufacturer’s protocols. The quality and quantity of the fungal genomic DNA obtained were assessed using an MD-1000 Nanodrop spectrophotometer (NanoDrop Technologies, Wilmington, DE, USA). The concentrations of the extracted DNA samples varied, so each sample was adjusted to a consistent working concentration of 100 ng/µL by calculating the necessary dilutions with nuclease-free water using the formula C₁V₁ = C₂V₂. This standardized DNA was then utilized as the template for PCR amplification.

Molecular identification of the representative isolates (based on host cultivar, site of isolation, and isolate aggressiveness) was conducted through DNA amplification and sequencing of a dataset comprising multiple loci, including the partial translation elongation factor 1-alpha (*tef1*), and the partial β-tubulin (*tub2*) gene as well as nuclear ribosomal internal transcribed spacer (ITS) region, and the partial 28S large subunit RNA (LSU) regions. The primers employed for this study were ITS1 and ITS4 [29] for the ITS region, LSU-R0R and LSU-R5 [30] for the LSU, EF1-728F [31] and EF1-2218R [32] for *tef1*, as well as Btub2Fd and Btub4Rd [33] for *tub2*. A Peltier thermal cycler (Bio-Techne, Minneapolis, MN, USA) was used to carry out the PCR amplifications. The thermal cycling protocols for PCR amplification of the ITS, LSU, *tef1*, and *tub2* genes are outlined in Table 1. Finally, 5 µL of each PCR amplification product was run on 1% agarose gels, stained with 0.05% ethidium bromide, to confirm successful amplification. The PCR products were then sequenced using the original amplification primers through a dye terminator cycle (Codon Genetic Group, Tehran, Iran).

**Table 1:** PCR programs were used in this study.

Gene^1^	Number of Cycles	Initial Denaturation	Denaturation	Annealing	Extension	Final Extension
ITS	35	95 (180)^2^	95 (15)	55 (30)	72 (60)	72 (600)
LSU	35	95 (180)	95 (15)	48 (30)	72 (60)	72 (600)
*tef1*	35	94 (120)	95 (45)	56 (60)	72 (60)	72 (600)
*tub2*	35	95 (120)	95 (90)	62 (60)	72 (45)	72 (600)

^1^ITS: internal transcribed spacers 1 and 2 and 5.8S rRNA gene of rDNA; LSU: partial 28S large subunit RNA gene; *tef1*: partial translation elongation factor 1–alpha gene; *tub2*: partial β-tubulin gene. ^2^ Temperature, °C (time, s).

### Phylogenetic analyses

DNA sequences generated in this study were initially processed using BioEdit v. 7.0.9.0 [34] for quality assessment and trimming of ambiguous bases. Preliminary taxonomic identification of isolates was performed via BLASTn (nucleotide-versus-nucleotide comparison) queries against the NCBI GenBank database. For multilocus phylogenetic reconstruction, four gene regions (ITS, LSU, *tef1*, and *tub2*) were analyzed. Reference sequences from NCBI GenBank (S2 Table) were aligned with newly generated sequences using the MAFFT v. 7 (http://mafft.cbrc.jp/alignment/server/index.html) [35]. Alignments were manually refined in MEGA v. 11.0.13 [36] to correct misaligned regions caused by indels, a critical step omitted in the original description. Partition homogeneity tests on the combined nuclear gene alignments were conducted with PAUP* 4.0a136 [37], employing 100 replicates and a heuristic search strategy. For the reconstruction of phylogenetic trees, Bayesian inference analyses were carried out for individual loci (ITS, LSU, *tef1*, and *tub2*) as well as for concatenated ITS, *tef1*, and *tub2* genes using MrBayes 3.1 [38], implemented in TrEase [39]. Phylogenetic analyses were performed for 1,000,000 generations under the GTR + G + I nucleotide substitution model, with 30% of the initial trees discarded as burn-in. Maximum Likelihood (ML) analysis was executed in RAxML (Stamatakis, 2014) under the GTR-GAMMA model (as default in TrEase), with 1,000 bootstrap replicates to assess node support. Nodes with bootstrap support (BS) ≥70% were typically considered well-supported [40]. Phylogenetic trees were visualized, edited, and annotated using MEGA v. 11.0.13. Novel sequence data generated in this study have been deposited in GenBank (http://www.ncbi.nlm.nih.gov/genbank).

#### Nomenclature.

In addition, new names contained in this work have been submitted to MycoBank from where they will be made available to the Global Names Index. The unique MycoBank number can be resolved and the associated information viewed through any standard web browser by appending the MycoBank number contained in this publication to the prefix http://www.mycobank.org/MB/. The online version of this work is archived and available from the following digital repositories: PubMed Central and LOCKSS.

#### Pairwise homoplasy index (PHI) analyses.

To evaluate the potential for historical recombination, we performed a pairwise homoplasy index (PHI) test [41] on a concatenated sequence alignment using SplitsTree v. 6.0.0 [42]. The dataset comprised 14 taxa, including the newly generated isolates and closely related species, and a 1,445 bp sequence alignment of the ITS, *tub2*, and *tef1* genes. A neighbor-net network [43,44] was inferred from a Hamming distance matrix to visualize phylogenetic conflict [45]. A significant *P*-value (*P* < 0.05) rejects the null hypothesis of strictly clonal evolution, providing evidence for recombination. The robustness of the network splits was assessed with 1,000 bootstrap replicates [46].

### Pathogenicity assessments

#### Initial pathogenicity test and isolates aggressiveness on fig detached shoots.

To establish pathogenicity and compare aggressiveness among *Thyrostroma* sp. isolates, one-year-old healthy-looking fig shoots (*F. carica* ‘Sabz’) shoots (mean diameter: 1.0 ± 0.1 cm, length: 25 ± 2 cm) were aseptically collected in June 2024 from a commercial orchard. Surface sterilization was performed by sequential immersion in 70% ethanol (1 min), 1% sodium hypochlorite solution (NaOCl; 3 min), and three rinses with sterile distilled water for one min each, followed by air-drying under a laminar flow hood. For artificially inoculation, five-millimeter mycelial plugs were excised from the actively growing margins of 14-day-old PDA cultures (incubated at 25°C in darkness) of each isolate (n = 31 isolates). A sterile scalpel was used to create uniform wounds (5 mm diameter × 2 mm depth) through the bark to the cambium at mid-length positions on shoots. Plugs were placed mycelium-side down on wounds, secured with Parafilm^®^ (Bemis Packaging, USA), and sealed at the upper end with paraffin wax to prevent desiccation. Control shoots received sterile PDA plugs.

The experiment followed a Completely Randomized Design (CRD) with three biological replicates (shoots) per isolate and control. Inoculated shoots were vertically suspended in 500 mL Erlenmeyer flasks containing 100 mL sterile distilled water (SDW) to maintain humidity and incubated for 10 days in a controlled-environment chamber (25 ± 1°C, 16-h light/8-h dark). External lesion length (LL) and width (LW) were measured on bark surfaces with a digital caliper (Insize^®^, China, 0.01 mm precision), while upward (UILP) and downward (DILP) internal progressions were assessed after bark removal. Koch’s postulates were confirmed by culturing surface-sterilized (1% NaOCl) wood fragments from lesion margins on PDA, and re-isolated fungi were morphologically compared to original isolates.

Statistical analyses were conducted using SAS 9.4 (SAS Institute Inc.) and R 3.4.0 (R Core Team, 2017). Data normality for the four pathogenicity traits (LL, LW, UILP, and DILP) was confirmed using the Shapiro–Wilk test. A generalized linear model (GLM) followed by one-way analysis of variance (ANOVA) was used to evaluate the isolates’ aggressiveness. Pairwise comparisons between isolates were performed using Tukey’s HSD (*P* < 0.05). To further understand the levels of aggressiveness of isolates based on the four pathogenicity traits, Principal Component Analysis (PCA) was conducted with R version 3.4.0 (http://www.r-project.org/). Additionally, to represent the severity of each pathogenicity trait in the assessment of isolate aggressiveness, heatmaps were created using the CIMMiner online tool hosted by the Genomics and Pharmacology Facility (discover.nci.nih.gov). The severity percentages for each pathogenicity trait were used to generate the heatmap.

#### Inoculation of one-year-old fig saplings.

The pathogenicity of the most aggressive fungal isolate was evaluated on one-year-old saplings of *Ficus carica* ‘Sabz’, a commercially important cultivar. Three inoculation methods were employed: (1) stem inoculation using mycelial plugs, (2) stem inoculation with a spore suspension, and (3) bud inoculation with a spore suspension (as described below in the following section). After inoculation, saplings were maintained in a greenhouse for four months under a 12-hour light/12-hour dark photoperiod and controlled temperatures (20–35 °C) in a completely randomized design, with three biological replicates (individual saplings) per treatment. Soil moisture was maintained at field capacity through regular watering. To fulfill Koch’s postulates, wood tissue fragments from the margins of symptomatic lesions were surface-sterilized and cultured on PDA. Emerging fungal colonies were subcultured, and the inoculated isolate was confirmed through morphological characterization.

#### Wood stem inoculation using mycelial plugs.

Sapling wood stems were inoculated by removing a 1 cm diameter circular section of bark and phloem with a sterile cork borer. Mycelial plugs (5 mm diameter × 3 mm thickness), taken from the growing edge of a 14-day-old fungal culture on PDA, were carefully placed into the exposed wounds. The inoculation sites were then covered with Parafilm to maintain humidity and prevent contamination. Negative controls were prepared similarly, but with sterile agar plugs instead of fungal plugs. Four months after inoculation, the following parameters were measured: lesion length (LL) and lesion width (LW) on the bark surface; upward internal lesion progression (UILP); downward internal lesion progression (DILP) after bark removal; and lesion depth (LD) from transverse sections of the inoculated stem segment using a digital caliper.

#### Wood stem and bud inoculation using the injection of spore suspension.

The most aggressive isolate of the pathogen was cultured on PDA plates and incubated at 20 ± 5 °C with a 12-hour light/12-hour dark photoperiod for 14 days, successfully inducing sporodochia formation. After development, individual structures were aseptically transferred using a sterile needle into a 1.5 mL Eppendorf tube containing 1 mL of sterile distilled water. The suspension was homogenized by vortexing and then filtered through double-layered sterile cheesecloth to remove mycelial debris. The conidial concentration was quantified with a hemocytometer and adjusted to 1 × 10^6^ conidia/mL using sterile distilled water. For stem inoculation, 2 mm diameter by 5 mm deep holes were drilled into fig wood stems (minimum diameter of 1 cm at the inoculation site) using a sterilized power drill bit. Bud inoculation sites were prepared by drilling holes with a diameter of 2 mm and a depth of 3 mm, located 5 cm below the apical bud. A 100 μL aliquot of the conidial suspension was injected into each wound using a sterile micropipette. Inoculation sites were immediately sealed with Parafilm to prevent contamination and desiccation [47]. Control saplings received mock inoculations with 100 μL of sterile distilled water [48]. Forty days after inoculation, longitudinal sections of stems and buds were examined to quantify symptom progression. The upward (UVP) and downward (DVP) vascular progression of necrosis/discoloration was measured from the inoculation point using a calibrated digital caliper.

#### Inoculation of leaves and fruit of the fig tree.

Healthy young leaves and fruits were collected from *Ficus carica* ‘Sabz’ trees and prepared as follows: samples were washed under running tap water, surface-sterilized in 0.5% NaOCl for 5 minutes, rinsed three times with sterile distilled water, and air-dried on sterile filter paper. For leaf inoculation, 2 × 2 mm wounds were created on the abaxial surface using sterile toothpicks. For fruits, 2 mm diameter × 3 mm deep wounds were made with a flame-sterilized cork borer. Mycelial plugs from a 14-day-old culture of the aggressive isolate were aseptically transferred to wounds: 3 × 3 mm plugs (abaxial side down) for leaves and 2 mm diameter plugs for fruits. Control samples received sterile PDA plugs without mycelium [11]. Inoculated tissues were placed in sterilized transparent containers lined with moist sterile cotton (hydrated with 5 mL sterile water). The containers were incubated at 25 ± 4°C under a 12-h photoperiod. Symptom progression (lesion diameter, and necrosis) was recorded at 5-day intervals over 15 days using digital calipers. To fulfill Koch’s postulates, 5 × 5 mm tissue segments from lesion margins were surface-sterilized (70% ethanol, 30 s), plated on PDA, and incubated at 25°C. Re-isolated fungi were identified morphologically via light microscopy (conidiophore structure, and conidial dimensions) and compared to the inoculated isolate.

## Results

### Disease symptoms and fungal isolates

**Fig 1:**
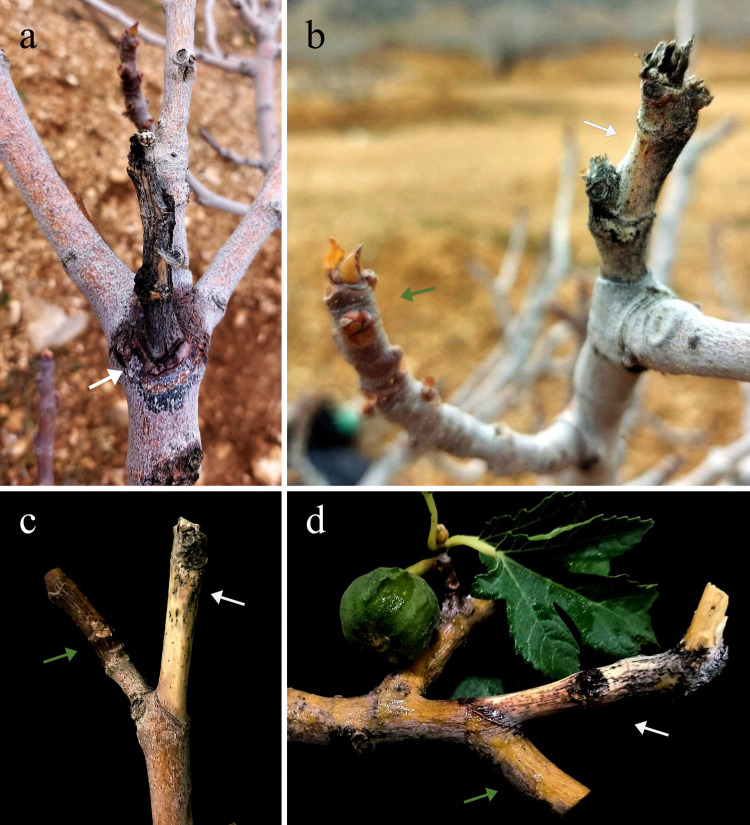
Symptoms of twig dieback induced by *Thyrostroma parviniae* sp. nov. on mature branches (a & c) and newly formed branches (b & d) of fig trees in Fars Province, Iran. Green arrows indicate healthy young branches, while white arrows delineate the necrotic tissues and symptoms of twig dieback.

Our 2023–2024 surveys in selected fig-growing regions of Fars Province revealed a marked increase in branch and twig dieback symptoms compared to earlier studies conducted from 2019 to 2022 [20,47,48]. Infected trees exhibited dieback on both mature and newly formed branches, followed by foliar yellowing, premature defoliation, and eventual mortality of main branches. Symptom progression initiated in young shoots, advancing toward larger branches without evidence of mechanical damage or pruning wounds (Fig 1). Cross-sectional analysis of symptomatic tissue confirmed extensive vascular necrosis and wood discoloration.

From these surveys, a total of 27 symptomatic fig trees exhibiting twig dieback were sampled (2–4 samples comprising both one-year-old and mature branches from each tree) across orchards in Fars Province. These samples yielded 152 fungal isolates associated with twig dieback. Morphological identification classified the majority as ascomycetes within the families *Bionectriaceae*, *Didymellaceae*, and *Pleosporaceae*. A subset of isolates (n = 31) belonged to the *Dothidotthiaceae* family. All *Dothidotthiaceae* isolates exhibited uniform cultural and morphological characteristics (including colony morphology, conidiomata structure, and conidial dimensions) and were thus considered as a morphotype. Of these, 11 were isolated exclusively from necrotic vascular tissue and 20 co-occurring with *Didymellaceae* and *Pleosporaceae* species. The *Dothidotthiaceae* isolates were recovered from two economically important cultivars: dry fig ‘Sabz’ and fresh fig ‘Siah’, sampled across four regions in Fars Province, including Estahban (6 isolates), Ij (13 isolates), Qalat (4 isolates), and Shiraz (8 isolates). Of the 31 isolates recovered, 22 were obtained from one-year-old branches and 9 from mature branches (Table 2).

**Table 2:** Information of *Thyrostroma parviniae* sp. nov. isolates collected from symptomatic fig trees in Fars Province, Iran.

Isolate code	Location	Host	Date	Type of sample	Symptom of sample^*^	Latitude	Longitude	Irrigation type	Collector
S73-55	Estahban	*Ficus carica* ‘Sabz’	21-May-2024	One-year-old branch	BD & BN	29.12046	53.99388	Rainfed	H. Negahban
S73-46	Estahban	*F. carica* ‘Sabz’	21-May-2024	One-year-old branch	BD & BN	29.12046	53.99388	Rainfed	H. Negahban
S72-45	Estahban	*F. carica* ‘Sabz’	21-May-2024	Mature branch	BD & IWD	29.12046	53.99388	Rainfed	H. Negahban
S72-44	Estahban	*F. carica* ‘Sabz’	21-May-2024	Mature branch	BD & IWD	29.12046	53.99388	Rainfed	H. Negahban
S65-42	Estahban	*F. carica* ‘Sabz’	21-May-2024	One-year-old branch	BD &BN	29.12046	53.99388	Rainfed	H. Negahban
S63-43	Estahban	*F. carica* ‘Sabz’	21-May-2024	One-year-old branch	BD & BN	29.12046	53.99388	Rainfed	H. Negahban
QSi2-13	Qalat	*F. carica* ‘Siah’	30-Oct-2023	Mature branch	BD & IWD	29.82341	52.32096	Rainfed	H. Negahban
QSi2-12	Qalat	*F. carica* ‘Siah’	30-Oct-2023	Mature branch	BD & IWD	29.82341	52.32096	Rainfed	H. Negahban
QSi2-11	Qalat	*F. carica* ‘Siah’	30-Oct-2023	Mature branch	BD & IWD	29.82341	52.32096	Rainfed	H. Negahban
QSi2-10	Qalat	*F. carica* ‘Siah’	30-Oct-2023	Mature branch	BD & IWD	29.82341	52.32096	Rainfed	H. Negahban
S87-54	Ij	*F. carica* ‘Sabz’	21-May-2024	One-year-old branch	BD & BN	29.02267	54.24054	Rainfed	H. Negahban
S87-53	Ij	*F. carica* ‘Sabz’	21-May-2024	One-year-old branch	BD & BN	29.02267	54.24054	Rainfed	H. Negahban
S87-52	Ij	*F. carica* ‘Sabz’	21-May-2024	One-year-old branch	BD &BN	29.02267	54.24054	Rainfed	H. Negahban
S87-51	Ij	*F. carica* ‘Sabz’	21-May-2024	One-year-old branch	BD & BN	29.02267	54.24054	Rainfed	H. Negahban
S87-41	Ij	*F. carica* ‘Sabz’	21-May-2024	One-year-old branch	BD & BN	29.02267	54.24054	Rainfed	H. Negahban
S87-40	Ij	*F. carica* ‘Sabz’	21-May-2024	One-year-old branch	BD & BN	29.02267	54.24054	Rainfed	H. Negahban
S84-57	Ij	*F. carica* ‘Sabz’	21-May-2024	Mature branch	BD	29.02267	54.24054	Rainfed	H. Negahban
S84-56	Ij	*F. carica* ‘Sabz’	21-May-2024	Mature branch	BD	29.02267	54.24054	Rainfed	H. Negahban
S83-50	Ij	*F. carica* ‘Sabz’	21-May-2024	One-year-old branch	BD & BN	29.02267	54.24054	Rainfed	H. Negahban
S83-49	Ij	*F. carica* ‘Sabz’	21-May-2024	One-year-old branch	BD & BN	29.02267	54.24054	Rainfed	H. Negahban
S83-48	Ij	*F. carica* ‘Sabz’	21-May-2024	One-year-old branch	BD & BN	29.02267	54.24054	Rainfed	H. Negahban
S83-47	Ij	*F. carica* ‘Sabz’	21-May-2024	One-year-old branch	BD & BN	29.02267	54.24054	Rainfed	H. Negahban
S73-58	Ij	*F. carica* ‘Sabz’	21-May-2024	Mature branch	BD & BN	29.02267	54.24054	Rainfed	H. Negahban
DF12	Shiraz	*F. carica*	03-May-2024	One-year-old branch	TD & BN	29.70774	52.40168	Rainfed	F. Moradi
DF09	Shiraz	*F. carica*	03-May-2024	One-year-old branch	TD & BN	29.70774	52.40168	Rainfed	F. Moradi
DF07	Shiraz	*F. carica*	03-May-2024	One-year-old branch	TD & BN	29.70774	52.40168	Rainfed	F. Moradi
DF06	Shiraz	*F. carica*	03-May-2024	One-year-old branch	TD & BN	29.70774	52.40168	Rainfed	F. Moradi
DF05	Shiraz	*F. carica*	03-May-2024	One-year-old branch	TD & BN	29.70774	52.40168	Rainfed	F. Moradi
DF03	Shiraz	*F. carica*	03-May-2024	One-year-old branch	TD & BN	29.70774	52.40168	Rainfed	F. Moradi
DF02	Shiraz	*F. carica*	03-May-2024	One-year-old branch	TD & BN	29.70774	52.40168	Rainfed	F. Moradi
DF01	Shiraz	*F. carica*	03-May-2024	One-year-old branch	TD & BN	29.70774	52.40168	Rainfed	F. Moradi

* BD: Branch dieback; BN: Bud necrosis; IWD: Internal wood discoloration; TD: Twig dieback.

### Molecular identification and phylogenetic analyses

BLASTn comparisons of the nuclear ribosomal LSU region for isolate QSi2-10 showed 100% similarity and coverage with *Thyrostroma cornicola* (CBS141280; NG_063940) and 100% similarity (98% coverage) with *T. celtidis* (MFLUCC 16-1186; MK751822). For the ITS region, isolates QSi2-10, S83-47, and S73-46 exhibited 100% similarity and coverage with *T. cornicola* (CBS141280; NR_154514). Analysis of the protein-coding *tef1* locus revealed 99.34% similarity (100% coverage) between QSi2-10 and *T. celtidis* (MFLUCC 16-1186; MK908022), 98.85% similarity (100% coverage) with *T. styphnolobii* (MFLUCC 16-1160; MK908026), and 98.68% similarity (100% coverage) with *T. moricola* (MFLU 16-1795; MK908023). The *tub2* sequences displayed 97.97% similarity (100% coverage) to *T. celtidis* (MFLUCC 16-1186; MK933791), 96.58% similarity (99% coverage) to *T. moricola* (MFLU 16-1795; MK933792), and 96.27% similarity (100% coverage) to *T. styphnolobii* (MFLUCC 16-1160; MK933795) (S2 Table).

**Fig 2:**
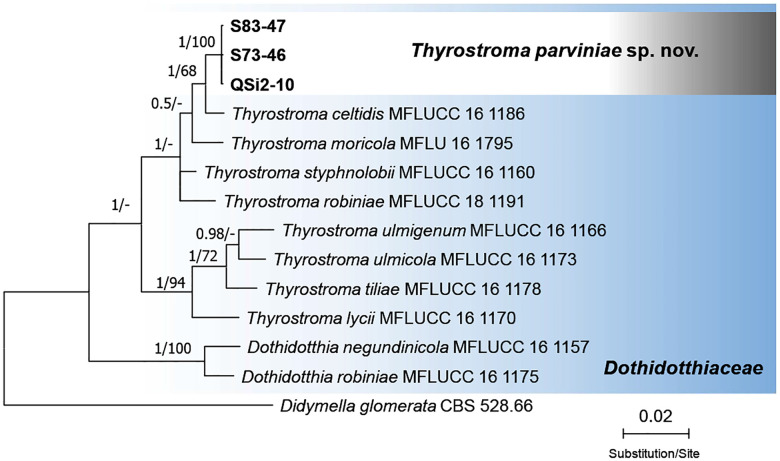
Phylogenetic position of *Thyrostroma parviniae* sp. nov. from twig dieback symptoms of fig trees sampled in Fars Province, Iran. The Bayesian Inference (BI) tree illustrates relationships among 10 species in *Dothiodotthiaceae* based on ITS (internal transcribed spacers 1 and 2 and 5.8S gene of rDNA), *tef1* (translation elongation factor 1-α), and *tub2* (β-tubulin) sequences. Numbers on the nodes represent Bayesian posterior probability values (BI–PP) followed by Maximum Likelihood bootstrap values (ML–BS). Branches with BI–PP = 1/ML–BS = 100 are considered fully supported. The tree was rooted to *Didymella glomerata* (CBS 528.66). QSi2-10 = ex-type = CBS 154728. Isolates retrieved from infected fig trees in Iran are indicated in bold.

Sequence alignments of the four nuclear loci yielded the following lengths: 709 nucleotides for LSU, 470 for ITS, 607 for *tef1*, and 295 for *tub2*. Comparative sequence analysis identified 13 conserved polymorphisms across the concatenated alignment, with three in ITS, four in *tef1*, and six in *tub2* (S3 Table). No diagnostic polymorphisms were detected in the LSU region (S4 Fig). Phylogenetic reconstruction of the concatenated ITS, *tef1*, and *tub2* datasets resolved all fig-derived isolates into a distinct, strongly supported monophyletic clade (ML bootstrap = 100%; BI posterior probability = 1.0), sister to *T. celtidis* (Fig 2).

Combined with morphological evidence, these molecular data, particularly the fixed polymorphisms in *tef1* and *tub2* and the strongly supported monophyly, support the recognition of the fig-associated lineage as a novel species, herein described as *Thyrostroma parviniae* sp. nov.

#### Pairwise homoplasy index (PHI) analyses.

The pairwise homoplasy index (PHI) test yielded a significant result (*P* = 0.036), indicating evidence of historical recombination within the concatenated alignment. Despite this detectable recombination, the isolates of *T. parviniae* sp. nov. formed a distinct and well-supported monophyletic clade (Bootstrap = 100), clearly separating them from other closely related species (S5 Fig).

### Taxonomy

*Thyrostroma parviniae* H. Negahban Z. Bolboli, & Mostowf. sp. nov.

MycoBank: MB859606

*Etymolog*y: This species is named in honor of Ms. Sharbanoo Parvin, a distinguished lecturer in plant pathology at Shiraz University, Iran.

*Typification*: Iran, Fars Province: Qalat Village, (29°82.341′ N − 52°32.096′ E), isolated from the symptom of dieback and internal wood discoloration on the mature branch of *Ficus carica* ‘Siah’, 30 October 2023, H. Negahban, stored in a metabolically inactive state, Herbarium Westerdijk Fungal Biodiversity Institute (CBS; Utrecht, The Netherlands); QSi2-10 = CBS 154728, ex-holotype cultures; GenBank: LSU = PV742616; ITS = PV742613; *tef1* = PV750644; *tub2* = PV763142.

**Fig 3:**
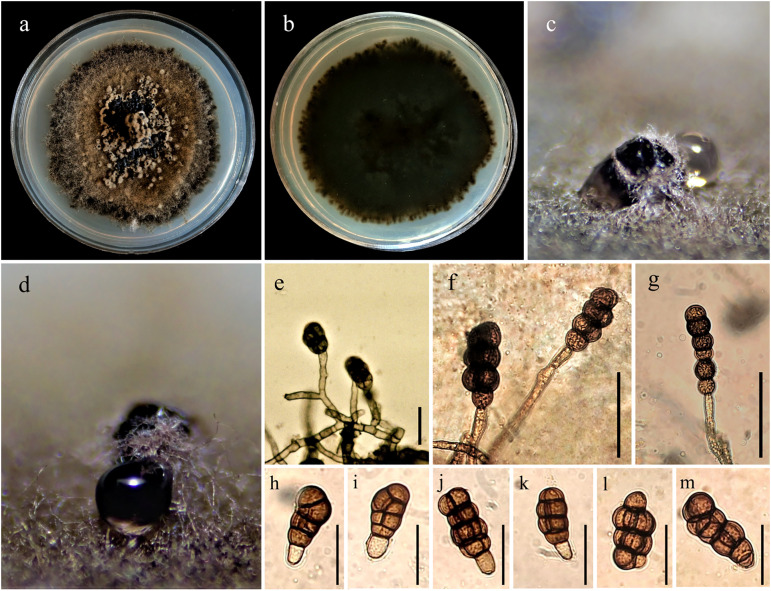
Colony morphology and microscopical structures of *Thyrostroma parviniae* sp. nov. on potato dextrose agar (PDA) at 20 °C, after 21 days. Obverse **(a)** and reverse **(b)** side views of the colony. Sporodochia on PDA **(c & d)**. Conidia attached to conidiogenous cells **(e–k)**. Conidia **(l & m)**. Scale bars: 50 µm.

*Description*: Conidiophores (length: 30.1–73.7 µm) × (width: 4.2–9.7 µm) (av. 52.7 ± 8.2 × 6.8 ± 1.1 µm, n = 150), macronematous, cylindrical, septate, branched, hyaline to pale brown, smooth, arising from the basal stroma. Conidiogenous cells (length: 5.9–23.9 µm) × (width: 6.0–15.5 µm) (av. 12.4 ± 2.9 × 9.8 ± 1.9 µm, n = 150), holoblastic, cylindrical to subcylindrical, integrated, terminal. Conidia (length: 22.8–77.9 µm) × (width: 13.2–34.3 µm) (av. 44.0 ± 8.3 × 24.0 ± 3.6 µm, n = 150), acrogenous, straight or curved, variable in shape, cylindrical, clavate to subclavate, or ellipsoidal to pyriform, rough-walled, minutely echinulate, pale to dark brown, rounded at apex, uniform, 2–6 transverse septa, with 0–5 longitudinal septa, constricted at the septa (Fig 3e–m), (S4 Table).

*Colony morphology*: Colonies slow-growing, circular, initially flat with a velvety (velutinous) surface, becoming pulvinate (cushion-shaped) with age. Margin distinct, irregular to fimbriate. Coloration: Initially grayish across the entire colony (0–7 days), maturing to olivaceous-brown at the center and jet black at the periphery after 14–21 days; reverse dark olivaceous-gray to black. Aerial mycelium: dense, cottony to tufted, raised centrally. Reproductive structures: Black sporodochia develop sparsely across the colony surface after 2–3 weeks, transitioning from pulvinate (dome-shaped) to applanate (flattened) with age (Fig 3a–b).

*Growth rate* (radial extension, mean ± SD, n = 3, in darkness): Optimal at 20 °C: 1.94 ± 0.80 mm/day (reaching 25–30 mm diameter in 14 days), reduced at 35 °C: 0.17 ± 0.03 mm/day (reaching 2–3 mm diameter in 14 days). No growth observed at 40 and 5 °C.

*Other specimens examined (paratypes)*: Isolate S73-46: Iran, Fars Province: Estahban (29°12.046′ N − 53°99.388′ E) from the branch dieback on a one-year-old branch of *Ficus carica* ‘Sabz’, 21 May 2024, H. Negahban, GenBank: ITS = PV763143; *tef1* = PV750645; *tub2* = PV763143. Isolate S83-47 (CBS 154727): Iran, Fars Province: Ij (29°02.267′ N − 54°24.054′ E) from branch dieback symptom of a one-year-old branch of *Ficus carica* ‘Sabz’, 21 May 2024, H. Negahban, GenBank: ITS = PV742615; *tef1* = PV750646; *tub2* = PV763144.

*Note*: *Tyrostroma parviniae* sp. nov. shares a close phylogenetic relationship with *T. celtidis*, [22], however, they exhibit distinct morphological differences. Compared to *T. celtidis*, the newly described *T. parviniae* sp. nov. is characterized by greater lengths across key morphological features: conidia (22.8–77.9 µm vs. 27–48 µm), conidiogenous cells (5.9–23.9 µm vs. 9–18 µm), and conidiophores (30.1–73.7 µm vs. 25–51 µm). The conidia of *T. parviniae* sp. nov. exhibit greater morphological diversity, ranging from cylindrical, clavate, and subclavate to ellipsoidal and pyriform forms, with 2–6 transverse septa and 0–5 longitudinal septa. In contrast, *T. celtidis* conidia are primarily clavate to obpyriform, with 3–6 transverse septa and 2–5 longitudinal septa [22]. Additionally, the two species are genetically distinct, with fixed nucleotide differences in the *tef1* and *tub2* gene regions (S2 and S3 Figs), further supporting their taxonomic separation.

### Pathogenicity assessments

#### Initial pathogenicity test and isolates aggressiveness on fig detached shoots.

**Fig 4:**
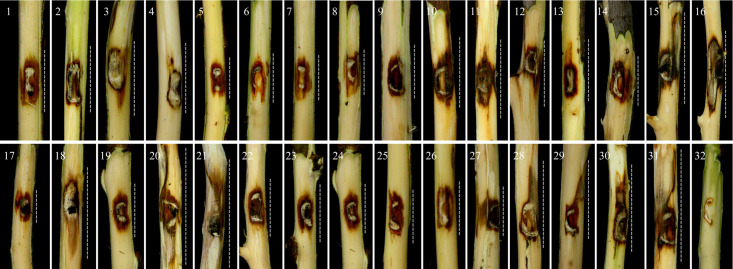
Aggressiveness levels of 31 *Thyrostroma parviniae* sp. nov. isolates causing wood necrosis and discoloration on fig detached shoots 10 days after inoculation. Isolates 1–31 correspond to: 1: DF01; 2: DF02; 3: DF03; 4: DF05; 5: DF06; 6: DF07; 7: DF09; 8: DF12; 9: QSi2-10; 10: QSi2-11; 11: QSi2-12; 12: QSi2-13; 13: S65-42; 14: S65-43; 15: S72-44; 16: S72-45; 17: S73-46; 18: S73-55; 19: S73-58; 20: S83-47; 21: S83-48; 22: S83-49; 23: S84-56; 24: S84-57; 25: S87-40; 26: S87-41; 27: S87-50; 28: S87-51; 29: S87-52; 30: S87-53; 31: S87-54; and 32: the negative control inoculated with non-colonized PDA. Dotted lines demarcate the boundaries of necrotic and discolored tissue.

**Fig 5:**
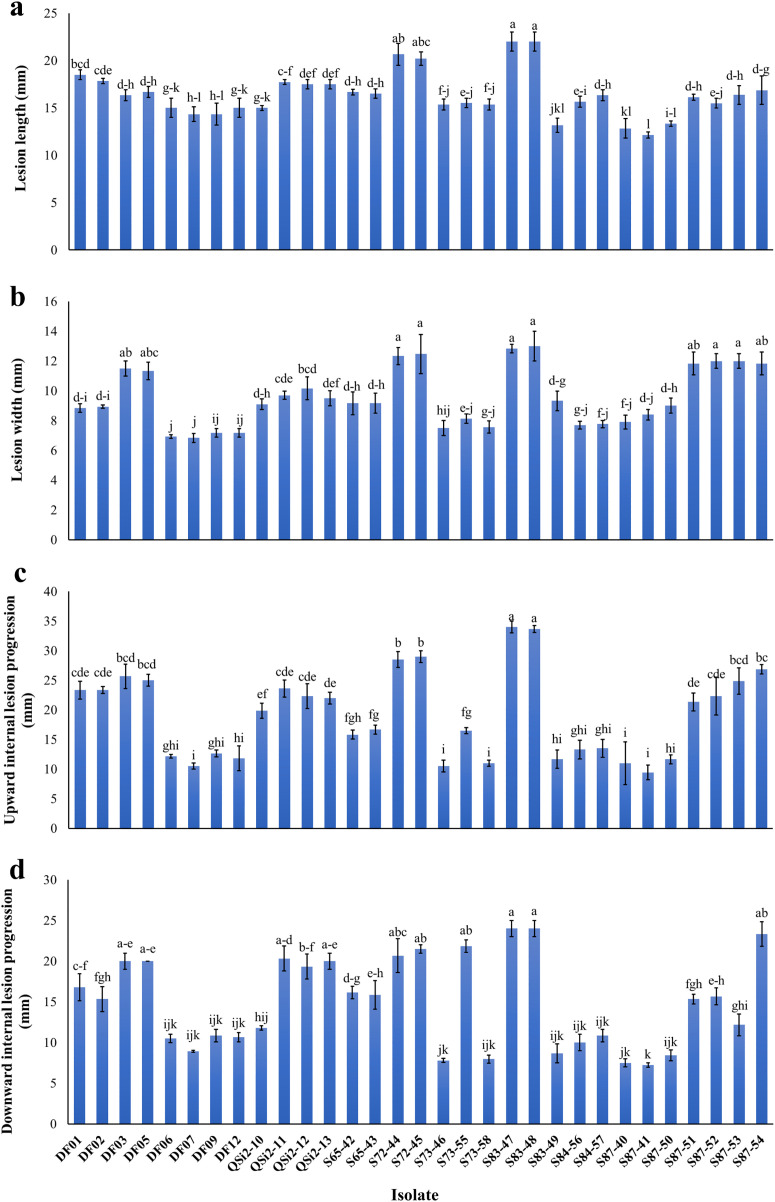
Pathogenicity traits assessed on fig detached shoots inoculated with 31 isolates of *Thyrostroma parviniae* sp. nov., 10 days after inoculation. The figure illustrates the average measurements of lesion length **(a)**, lesion width **(b)**, upward internal lesion progression **(c)**, and downward internal lesion progression **(d)**. Distinct lowercase letters denote statistically significant differences among isolates (*P* ≤ 0.05). Error bars represent standard deviation of three biological replicates (n = 3).

In pathogenicity assays on fig detached shoots, all 31 studied isolates induced necrotic lesions within 10 days after inoculation, compared to the controls (Fig 4). The successful re-isolation of tested isolates from inoculated detached shoots, coupled with their absence in control shoots, provided evidence supporting their pathogenicity. Analysis of variance (ANOVA) revealed significant differences (*P* < 0.0001) in pathogenicity traits, specifically LL, LW, UILP, and DILP, among the tested isolates (S5 Table). Post-hoc Tukey’s tests demonstrated significant variation in lesion development. Isolates S83-47 and S83-48 had an average LL of 22.0 ± 1.0 mm, while S87-41 exhibited a smaller mean of 12.13 ± 0.32 mm. Similarly, the average LW for S83-47 and S83-48 was 12.83 ± 0.29 mm and 13.0 ± 1.0 mm, respectively, compared to a narrower measurement of 6.83 ± 0.29 mm for DF07. Among the isolates analyzed, the mean UILP ranged from 9.43 ± 1.25 mm for S87-41 to 34.0 ± 1.0 mm for S83-47. Additionally, the mean DILP for symptoms ranged from 7.23 ± 0.25 mm for S87-41 to 24.00 ± 1.00 mm for both S83-47 and S83-48 (Figs 4 and 5).

**Fig 6:**
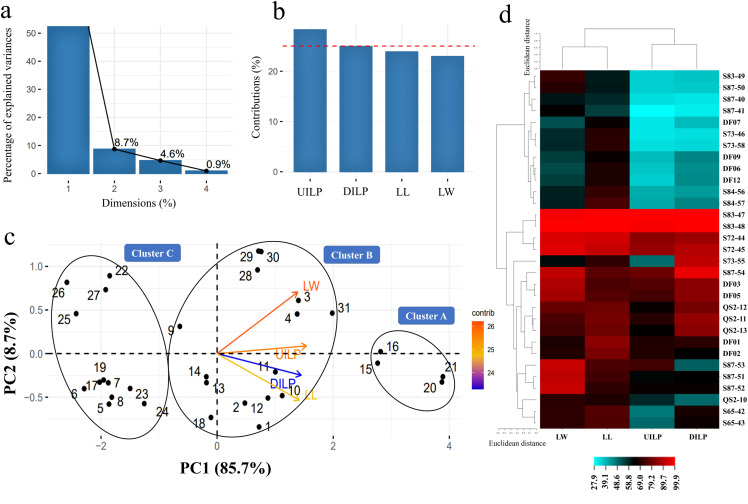
Principal Component Analysis (PCA) and heatmap of the aggressiveness assessment dataset of *Thyrostroma parviniae* sp. nov. isolates on detached fig shoots. **(a)** The percentage of explained variance for each of the four dimensions (principal components). **(b)** The bar chart displays the contribution percentage of each variable (pathogenicity traits), including lesion length (LL), lesion width (LW), upward internal lesion progression (UILP), and downward internal lesion length progression (DILP) in the first principal component. **(c)** The PCA biplot, including the first (PC1) and the second (PC2) principal components categorized the isolates into three groups based on the four pathogenicity traits: Cluster A, highly aggressive isolates (15: S72-44; 16: S72-45; 20: S83-47; 21: S83-48); Cluster B, moderately aggressive isolates (1: DF01; 2: DF02; 3: DF03; 4: DF05; 9: QSi2-10; 10: QSi2-11; 11: QSi2-12; 12: QSi2-13; 13: S65-42; 14: S65-43; 18: S73-55; 28: S87-51; 29: S87-52; 30: S87-53; 31: S87-54); and Cluster C, less aggressive isolates (5: DF06; 6: DF07; 7: DF09; 8: DF12; 17: S73-46; 19: S73-58; 22: S83-49; 23: S84-56; 24: S84-57; 25: S87-40; 26: S87-41; 27: S87-50). **(d)** The heatmap shows the percentages representing each pathogenicity trait’s severity were calculated using the formula: (value of each pathogenicity trait/maximum value in that trait) × 100.

Principal component analysis (PCA) of pathogenicity traits revealed that the first two principal components (PC1 and PC2) collectively accounted for 94.4% of the total variance, with PC1 contributing 85.7% and PC2 8.7% (Fig 6a). Among the analyzed traits, upward internal lesion progression (UILP) was identified as the primary driver of variation along PC1 (Fig 6b). Aggressiveness-based clustering segregated the isolates into three distinct clusters: Cluster A comprised highly aggressive isolates (S72-44, S72-45, S83-47, S83-48); Cluster B included moderately aggressive isolates (DF01, DF02, DF03, DF05, QSi2-10, QSi2-11, QSi2-12, QSi2-13, S65-42, S65-43, S73-55, S87-51, S87-52, S87-53, S87-54); and Cluster C consisted of less aggressive isolates (DF06, DF07, DF09, DF12, S73-46, S73-58, S83-49, S84-56, S84-57, S87-40, S87-41, S87-50) (Fig 6c). Additionally, heatmap analysis highlighted that isolates S83-47 and S83-48 exhibited the highest levels of four pathogenicity traits, corroborating the PCA findings (Fig 6d). As a result, the aggressive isolate S83-47 was selected for further investigation into its pathogenicity.

#### Inoculation of one-year-old fig saplings.

**Fig 7:**
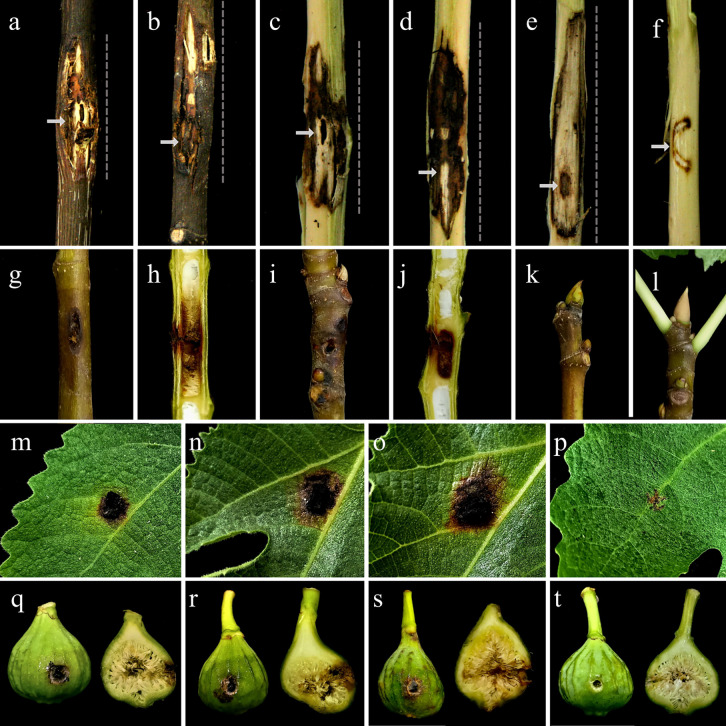
Pathogenicity of *Thyrostroma parviniae* sp. nov. (the most aggressive isolate, S83-47) on artificially inoculated one-year-old fig saplings, leaves, and fruits. Stem necrosis and wood discoloration induced by mycelial plug inoculation **(a–e)**. External symptoms (a–b, before bark removal) and internal necrosis and discoloration (c–e, after bark removal) on stems four months after inoculation. Internal stem discoloration following spore suspension inoculation **(g–j)**, woody stem (g) and its longitudinal section (h); young non-woody stem (i) and its longitudinal section (j), 40 days after inoculation. Bud symptoms 40 days after inoculation **(k–l)**: necrosis in spore-inoculated buds (k) versus asymptomatic sterile water-inoculated control (l). Leaf necrosis progression on spore-inoculated leaves **(m–o)** at five (m), 10 (n), and 15 (o) days after inoculation. Fruit symptom progression following spore inoculation **(q–s)**: early necrosis observed at 5 days after inoculation (q), progressive discoloration expansion by 10 days (r), and extensive tissue deterioration at 15 days (s). Negative controls **(f, p,** **t)**: Stem (f), leaf (p), and fruit (t) tissues inoculated with sterile potato dextrose agar (PDA) and sterile distilled water (l) remained asymptomatic throughout the observation period.

**Wood stem inoculation using mycelial plugs:** Pathogenicity evaluations conducted on wood stems of one-year-old fig saplings using mycelial plugs demonstrated that the most aggressive isolate (S83-47) induced disease symptoms four months after inoculation. Symptomatic saplings developed dark brown necrotic lesions radiating from the inoculation site on the main stem, accompanied by pronounced canker formation and extensive internal wood discoloration (Fig 7a–f). In contrast, control saplings inoculated with sterile agar plugs exhibited no disease progression beyond minor wound scars at the inoculation site. The re-isolation of inoculated fungus from inoculated wood stems, along with its absence in control tissues displaying minor discoloration, provided evidence supporting its role in inducing symptoms. The following pathogenicity characteristics were quantified: LL (av. 36.17 ± 0.80 mm), LW (av. 10.33 ± 0.76 mm), UILP (av. 21.67 ± 1.53 mm), DILP (av. 4.33 ± 2.08 mm), and LD (av. 2.42 ± 0.09 mm).

**Wood stem and bud inoculation via spore suspension injection:** Pathogenicity assays using conidial suspensions demonstrated that the isolate S83-47 induced internal discoloration in sapling wood stems and young buds within 40 days of inoculation (Fig 7g–l). Longitudinal sections of inoculated stems exhibited upward (UVP) and downward (DVP) vertical discoloration lengths of 23.87 ± 7.44 mm and 22.13 ± 9.21 mm, respectively. Inoculated buds showed significantly shorter UVP and DVP measurements (av. 9.03 ± 2.02 mm and 9.53 ± 2.83 mm, respectively). No symptoms were observed in control saplings treated with sterile water. The inoculated fungus was successfully reisolated from discolored stem and bud tissues, fulfilling Koch’s postulates.

#### Inoculation of leaves and fruit of the fig tree.

Pathogenicity assays on fig leaves and fruits revealed that the inoculated fungus caused necrotic lesions on both tissues. Inoculated leaves developed small, circular to irregular dark brown lesions with brown-yellowish margins within five days, expanding to 11.10 ± 2.58 × 9.8 ± 1.64 mm by day 15 (Fig 7m–p). Similarly, inoculated fruits displayed circular brown lesions starting 5 days after inoculation. Cross-sectional analysis showed that fungal infection caused superficial damage to internal cavities, contrasting with the asymptomatic internal tissue of control fruits (Fig 7q–t). The inoculated isolate of *T. parviniae* sp. nov. was successfully reisolated from lesions on both inoculated fruits and leaves, fulfilling Koch’s postulates.

## Discussion

This study documents the emergence of a novel fungal pathogen, *T. parviniae* sp. nov., which contributes to branch and twig dieback in the main fig production counties of Fars Province, Iran. This novel pathogen causes bud necrosis, branch dieback, and internal wood discoloration in commercially important dry (cv. ‘Sabz’) and fresh (cv. ‘Siah’) fig trees. While our extensive prior research focused on fig trunk and branch diseases [20,48,49], the distinct bud necrosis symptoms observed here, which is fundamentally different from previously documented cankers and diebacks symptoms, suggested the potential involvement of a novel pathogen. This new syndrome is distinguished by its target tissue (meristematic buds), initial symptom location (apices), and its acropetal, top-down disease progression. The convergence of unique morphological characteristics, multi-locus phylogenetic analysis, and the fulfillment of Koch’s postulates confirms both the novelty of this pathogen and its causal role in the disease.

Phylogenetic inference using LSU, ITS, *tef1*, and *tub2* loci placed the studied isolates in a monophyletic clade within *Thyrostroma* (*Dothiodotthiaceae*), sister to *T. celtidis*, yet genetically distinct. According to Index Fungorum (accessed 24 November 2025), the genus *Thyrostroma* contains 33 accepted species. Historically, a total of 35 scientific names have been published, representing 33 unique specific epithets [50]. Meanwhile, DNA sequences for 15 of these species are available in GenBank (accessed 24 November 2025). Notably, among the protein-coding genes, *tub2* exhibited the highest discriminatory power, revealing six single-nucleotide polymorphisms between *T. parviniae* sp. nov. and *T. celtidis* at distinct positions. This finding supports Senwanna et al. [22], who highlighted the usefulness of *tub2* for species delimitation within *Dothiodotthiaceae*, particularly for *Thyrostroma* spp. However, the current lack of *tub2* data on *Thyrostroma* spp. in public databases (*e.g.*, GenBank) remains a significant taxonomic constraint. We strongly advocate for future studies to prioritize *tub2* sequencing to refine species boundaries and uncover potential cryptic diversity within the genus.

Phylogenetically, *T. parviniae* sp. nov. is closely related to *T. celtidis*, indicating a recent divergence from a common ancestor. However, morphologically, *T. parviniae* sp. nov. differs from its closest relative, *T. celtidis*, by producing larger conidia and conidiophores, as well as exhibiting greater conidial polymorphism, cylindrical to pyriform vs. clavate-obpyriform in *T. celtidis*. Compared to other close relatives, *T. cornicola*, *T. moricola*, and *T. robiniae*, *T. styphnolobii*, [22,24], the isolates described here produce longer conidiophores and exhibit differences in septation patterns conidial septa. Notably, *T. parviniae* sp. nov. isolates also produce larger conidia than *T. cornicola* and *T. styphnolobii*. These consistent phenotypic distinctions, along with fixed genetic differences in *tef1* and *tub2*, strongly support its designation as a new species.

Our phylogenetic analyses robustly delineate the new isolates as a distinct, monophyletic lineage, strongly supporting their status as a separate species. However, the significant signal of recombination detected by the PHI test (P = 0.036) indicates a complex evolutionary history involving historical gene flow, this does not invalidate the proposed species boundary. Instead, we interpret this as evidence that ancestral recombination events likely preceded the speciation event. The formation of a cohesive, well-supported clade signifies that these isolates have subsequently evolved in genetic isolation, accumulating fixed differences that define them as a unique evolutionary lineage. The observed phylogenetic conflict thus reflects the lineage’s recombinant ancestry before its cladogenesis, rather than ongoing gene flow that would obscure species boundaries. This finding underscores that the detection of recombination is not incompatible with well-defined species delimitation but rather adds depth to our understanding of their evolutionary trajectory. Future studies with broader genomic data will enable a more comprehensive analysis.

Pathogenicity evaluation using two methods, mycelial plugs and spore suspensions, on one-year-old fig saplings demonstrated the ability of *T. parviniae* sp. nov. to induce disease symptoms. Our comprehensive assessment successfully fulfilled Koch’s postulates, confirming that this species causes brown lesions, limb blight, and internal wood discoloration in detached fig shoots, as well as in the stems and buds of one-year-old saplings. The bud necrosis symptoms induced by artificial inoculation closely mirrored those observed in infected fig orchards. Symptom development in young shoots without mechanical wounds under natural conditions, suggests potential airborne conidial dispersal or latent infection. Furthermore, our findings indicate optimal in vitro growth at 20 °C, implying that disease peaks are likely to coincide with spring and autumn conditions. Under climate warming, temperature-mediated pathogenicity could accelerate spring phenology [51], potentially extend infection windows and exacerbate yield losses. However, further investigation is needed to confirm these hypotheses regarding this emerging pathogen.

*Thyrostroma* species are globally distributed agents associated with stem canker and dieback on a wide range of hosts, including members of the *Cannabaceae*, *Cornaceae*, *Ephedraceae*, *Elaeagnaceae*, *Fabaceae*, *Malvaceae*, *Moraceae*, *Rosaceae*, *Solanaceae*, *Ulmaceae*, and *Vitaceae* families [22–26,52]. A review of the literature reveals a significant gap in studies applying Koch’s postulates to verify the pathogenic nature of the *Thyrostroma* isolates on these hosts. To date, only two studies have successfully demonstrated the pathogenicity of *Thyrostroma* spp. associated with canker symptoms on *Amygdalus scoparia* (syn. *Prunus scoparia* Spach) and *Vitis vinifera* [25,26]. The ability of various *Thyrostroma* species to colonize a wide range of host plants across different families likely suggests remarkable adaptability and potential for host range expansion of *T. parviniae* sp. nov. under favorable conditions. Further pathogenicity testing, including host range assessments and cultivar susceptibility evaluations of this new fig pathogen, as well as other *Thyrostroma* species recovered from symptomatic plant material, is strongly recommended to elucidate their host specificity and pathogenic potential.

Principal component analysis (PCA) of aggressiveness test results on detached shoots enabled the development of a scale for grouping isolate pathogenicity based on four disease characteristics, with upward internal lesion progression (UILP) as a key indicator. The clustering of isolates into highly, moderately, and less aggressive groups likely reflects genetic diversity within *T. parviniae* sp. nov. For example, the aggressive group may correlate with enhanced cell-wall-degrading exoenzymes, virulence factors in wood-decaying fungi [53]. Artificial inoculation of detached shoots has been previously validated as a low-cost, rapid, and effective method for assessing isolate aggressiveness [48,54,55].

Additionally, the pathogenicity assessment of *T. parviniae* sp. nov. indicates its potential to induce disease symptoms on fig leaves and fruits, potentially compromising fig yield and quality through the development of leaf and fruit spots. This observation aligns with previous studies confirming the pathogenic ability of another member of *Dothidotthiaceae*, *T. carpophilum* (syn. *Wilsonomyces carpophilus*), to cause shot hole disease in stone fruits, a significant disease associated with substantial damage [56,57]. The ability of *T. parviniae* sp. nov. to infect stems, buds, leaves, and fruits mirrors the broad tissue tropism of *T. carpophilum*. Notably, based on disease symptoms observed under natural conditions, it appears that *T. parviniae* sp. nov. does not require mechanical wounds for infection, suggesting that it employs appressoria or enzymatic digestion for host entry, which warrants further ultrastructural investigation.

This study successfully identified *T. parviniae* sp. nov*.*, a novel pathogenic fungal species within the *Dothidotthiaceae* family, as a significant contributor to twig dieback symptoms observed in Iranian fig orchards. Pathogenicity tests unequivocally demonstrated that isolates of *T. parviniae* sp. nov. can induce disease in detached fig shoots and saplings. While tree dieback and decline result from a complex interplay of biotic and abiotic factors, fungi often play a prominent role in canker and dieback diseases, leading to tree mortality across various species and geographic regions [58]. Moreover, environmental conditions, including climate change, significantly impact disease incidence by affecting overall plant health [59]. Notably, climate change influences the prevalence and behavior of canker/dieback-causing pathogens, particularly in relation to the dynamics of latent infections in diverse tree species [51,60]. Therefore, future research should prioritize comprehensive epidemiological studies that encompass the entire disease triangle, susceptible host, invasive pathogen, and conducive environmental conditions, to thoroughly understand and address tree mortality. Additionally, there is a pressing need for the development of rapid and accurate molecular detection methods for *T. parviniae* sp. nov. in both symptomatic and asymptomatic plant materials from fig nurseries and orchards. Investigating the potential host range and assessing the susceptibility of various fig cultivars to this pathogen will be vital for formulating effective management and control strategies.

## Supporting information

S1 Fig Phylogenetic position of *Thyrostroma parviniae* sp. nov. from twig dieback symptoms of fig trees sampled in Fars Province, Iran. The Bayesian Inference (BI) tree, rooted to outgroup taxa *Didymella glomerata* (CBS 528.66) and *Phoma herbarum* (CBS 615.75), illustrates relationships among 29 species in *Dothiodotthiaceae* based on ITS (internal transcribed spacers 1 and 2 and 5.8S gene of rDNA) sequences. Numbers on the nodes indicate Bayesian posterior probability values (BI–PP) followed by Maximum Likelihood bootstrap values (ML–BS). Branches with BI–PP = 1/ML–BS = 100 are considered fully supported. QSi2-10 = ex-type = CBS 154728. Isolates retrieved from infected fig trees in Iran are indicated in bold. Arrows represent the exact position of bootstrap values in the phylogenetic tree.
(TIF)

S2 Fig Phylogenetic position of *Thyrostroma parviniae* sp. nov. from twig dieback symptoms of fig trees sampled in Fars Province, Iran. The Bayesian Inference (BI) tree, rooted to outgroup taxa *Didymella glomerata* (CBS 528.66), illustrates relationships among 19 species in *Dothiodotthiaceae* based on tef1 (translation elongation factor 1-α) sequences. Numbers on the nodes represent Bayesian posterior probability values (BI–PP) followed by Maximum Likelihood bootstrap values (ML–BS). Branches with BI–PP = 1/ML–BS = 100 are considered fully supported. The tree is rooted to *Didymella glomerata* (CBS 528.66). QSi2-10 = ex-type = CBS 154728. Isolates retrieved from infected fig trees in Iran are indicated in bold. Arrows represent the exact position of bootstrap values in the phylogenetic tree.
(TIF)

S3 Fig Phylogenetic position of *Thyrostroma parviniae* sp. nov. from twig dieback symptoms of fig trees sampled in Fars Province, Iran. The Bayesian Inference (BI) tree, rooted to outgroup taxa *Didymella glomerata* (CBS 528.66) and *Phoma herbarum* (CBS 615.75), illustrates relationships among 10 species in *Dothiodotthiaceae* based on tub2 (β-tubulin) sequences. Numbers on the nodes represent Bayesian posterior probability values (BI–PP) followed by Maximum Likelihood bootstrap values (ML–BS). Branches with BI–PP = 1/ML–BS = 100 are considered fully supported. The tree is rooted to *Didymella glomerata* (CBS 528.66) and *Phoma herbarum* (CBS 615.75). QSi2-10 = ex-type = CBS 154728. Isolates retrieved from infected fig trees in Iran are indicated in bold.
(TIF)

S4 Fig Phylogenetic position of *Thyrostroma parviniae* sp. nov. from twig dieback symptoms of fig trees sampled in Fars Province, Iran. The Bayesian Inference (BI) tree, rooted to outgroup taxa *Didymella glomerata* (CBS 528.66) and *Phoma herbarum* (CBS 615.75), illustrates relationships among 28 species in *Dothiodotthiaceae* based on partial large subunit ribosomal RNA (LSU) gene sequences. Numbers on the nodes indicate Bayesian posterior probability values (BI–PP) followed by Maximum Likelihood bootstrap values (ML–BS). Branches with BI–PP = 1/ML–BS = 100 are considered fully supported. QSi2-10 = ex-type = CBS 154728. The isolate retrieved from infected fig trees in Iran is indicated in bold. Arrows represent the exact position of bootstrap values in the phylogenetic tree.
(TIF)

S5 Fig Pairwise homoplasy index (PHI) test using a neighbor-net network was inferred from a Hamming distance matrix to visualize phylogenetic conflict. The PHI test was conducted for *Thyrostroma parviniae* sp. nov. and its closely related species. The isolates of the new species are highlighted in a red box on the graph.
(TIF)

S1 Table Documented host plants of *Thyrostroma* species. (DOCX)

S2 Table Information of isolates included in the phylogenetic analysis in this study. (DOCX)

S3 Table Nucleotide differences across LSU, ITS, *tef1*, and *tub2* loci illustrating interspecific variation between *Thyrostroma parviniae* sp. nov. and related species. (DOCX)

S4 Table Morphometric measurements of conidia, conidiogenous cells and conidiophore from *Thyrostroma parviniae* sp. nov. isolates collected from infected fig trees in Fars Province, Iran. (DOCX)

S5 Table One-way Analysis of variance (ANOVA) results of four pathogenicity traits on one-year-old branches of fig trees inoculated with *Thyrostroma parviniae* sp. nov. (DOCX)
