# Supplementary figures and images for: Thyrostroma parviniae sp. nov., causing bud necrosis and branch dieback in fig trees from Iran
Source: PLoS One. 2026 Apr 8;21(4):e0341992. doi: 10.1371/journal.pone.0341992 (PMC13061225; doi:10.1371/journal.pone.0341992)

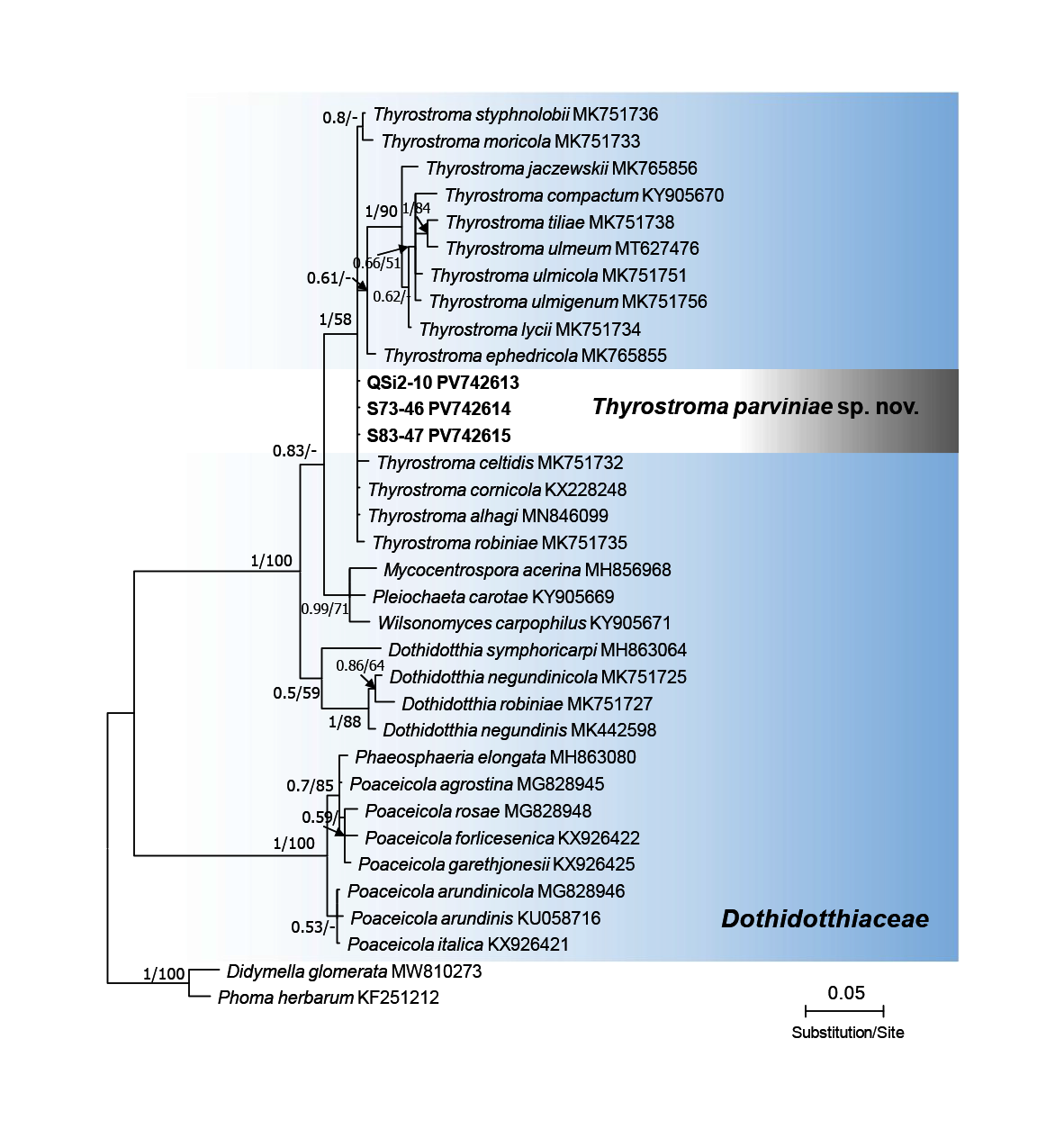

Supplement: S1 Fig — The Bayesian Inference (BI) tree, rooted to outgroup taxa Didymella glomerata (CBS 528.66) and Phoma herbarum (CBS 615.75), illustrates relationships among 29 species in Dothiodotthiaceae based on ITS (internal transcribed spacers 1 and 2 and 5.8S gene of rDNA) sequences. Numbers on the nodes indicate Bayesian posterior probability values (BI–PP) followed by Maximum Likelihood bootstrap values (ML–BS). Branches with BI–PP = 1/ML–BS = 100 are considered fully supported. QSi2-10 = ex-type = CBS 154728. Isolates retrieved from infected fig trees in Iran are indicated in bold. Arrows represent the exact position of bootstrap values in the phylogenetic tree. (TIF) [file pone.0341992.s001.tif]

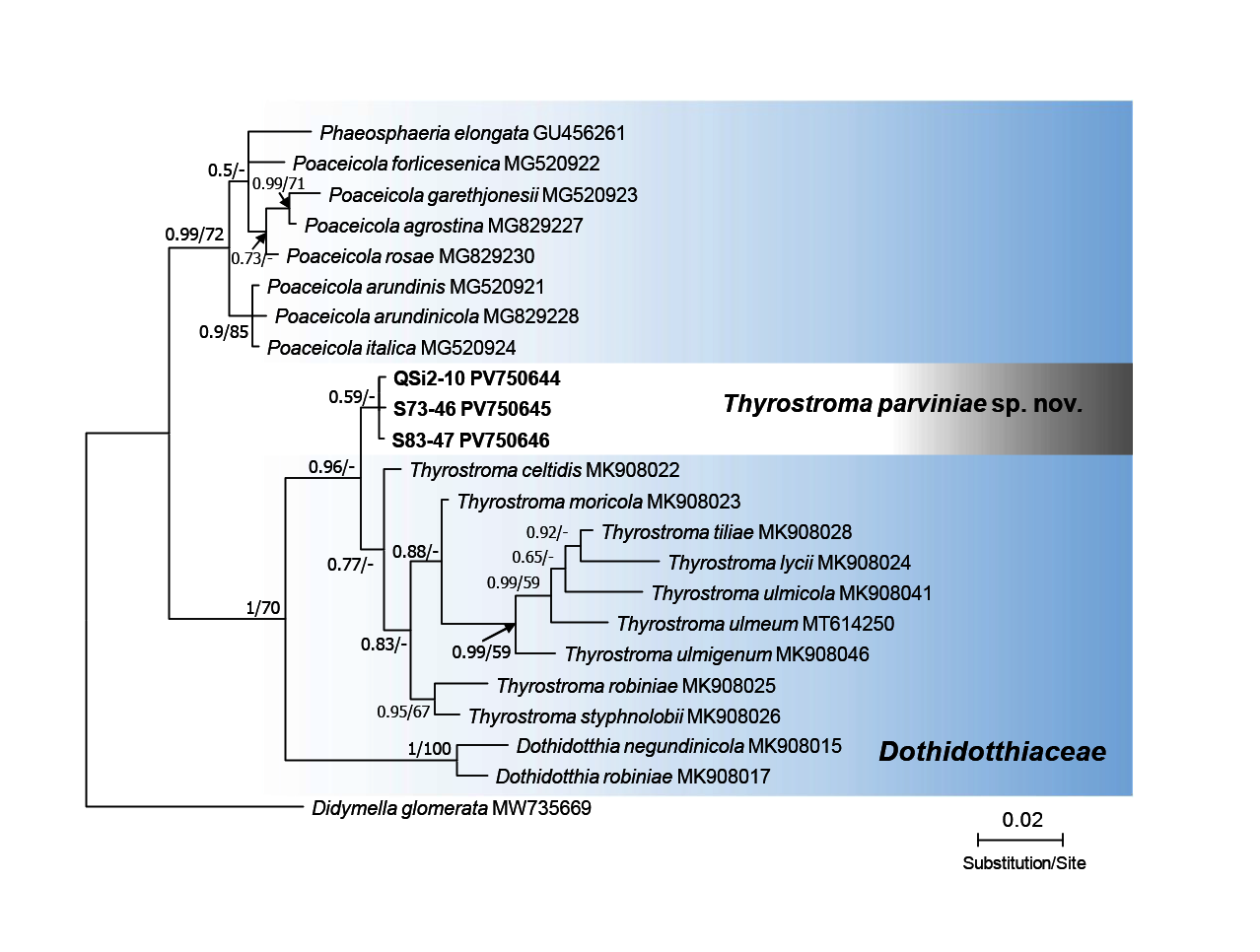

Supplement: S2 Fig — The Bayesian Inference (BI) tree, rooted to outgroup taxa Didymella glomerata (CBS 528.66), illustrates relationships among 19 species in Dothiodotthiaceae based on tef1 (translation elongation factor 1-α) sequences. Numbers on the nodes represent Bayesian posterior probability values (BI–PP) followed by Maximum Likelihood bootstrap values (ML–BS). Branches with BI–PP = 1/ML–BS = 100 are considered fully supported. The tree is rooted to Didymella glomerata (CBS 528.66). QSi2-10 = ex-type = CBS 154728. Isolates retrieved from infected fig trees in Iran are indicated in bold. Arrows represent the exact position of bootstrap values in the phylogenetic tree. (TIF) [file pone.0341992.s002.tif]

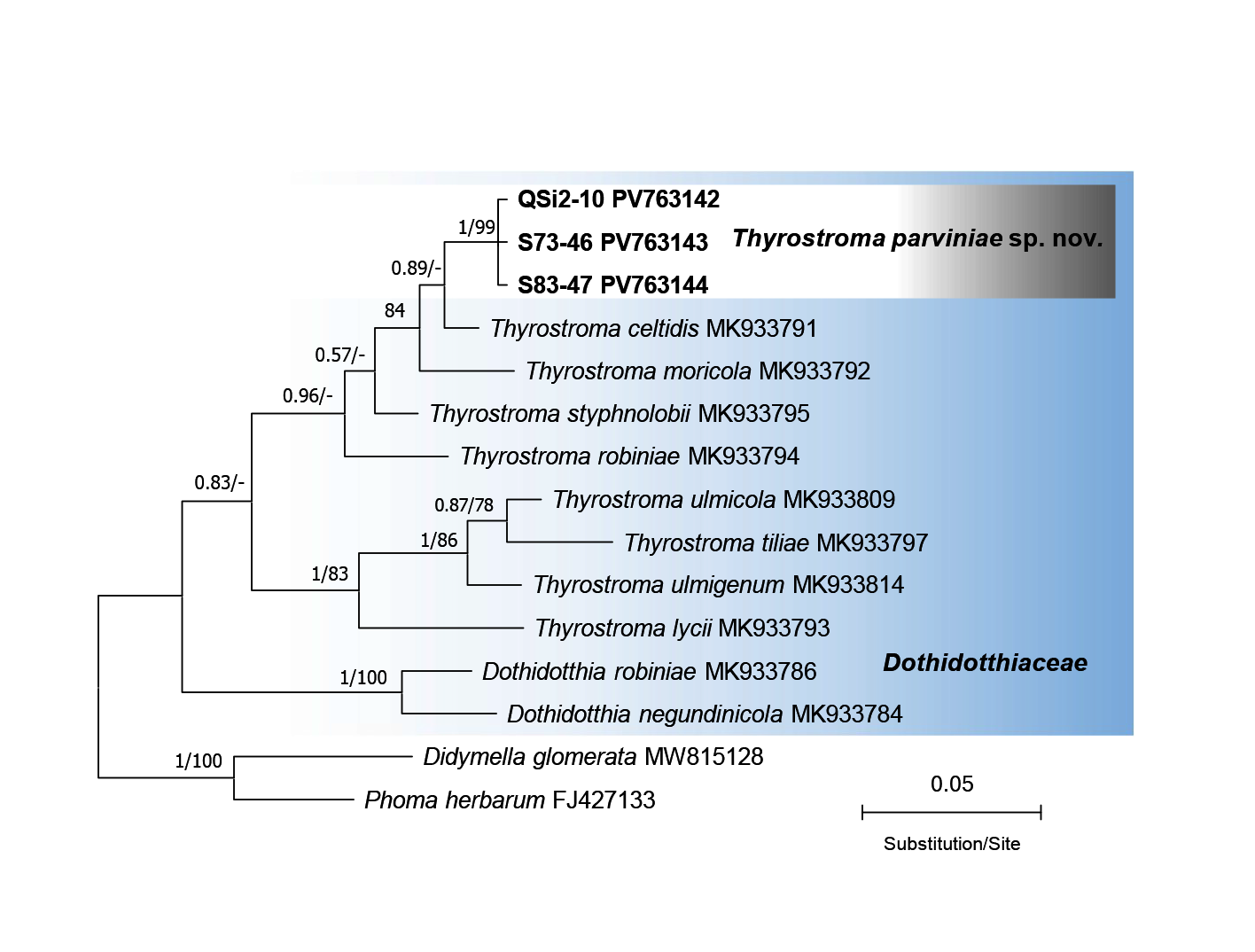

Supplement: S3 Fig — The Bayesian Inference (BI) tree, rooted to outgroup taxa Didymella glomerata (CBS 528.66) and Phoma herbarum (CBS 615.75), illustrates relationships among 10 species in Dothiodotthiaceae based on tub2 (β-tubulin) sequences. Numbers on the nodes represent Bayesian posterior probability values (BI–PP) followed by Maximum Likelihood bootstrap values (ML–BS). Branches with BI–PP = 1/ML–BS = 100 are considered fully supported. The tree is rooted to Didymella glomerata (CBS 528.66) and Phoma herbarum (CBS 615.75). QSi2-10 = ex-type = CBS 154728. Isolates retrieved from infected fig trees in Iran are indicated in bold. (TIF) [file pone.0341992.s003.tif]

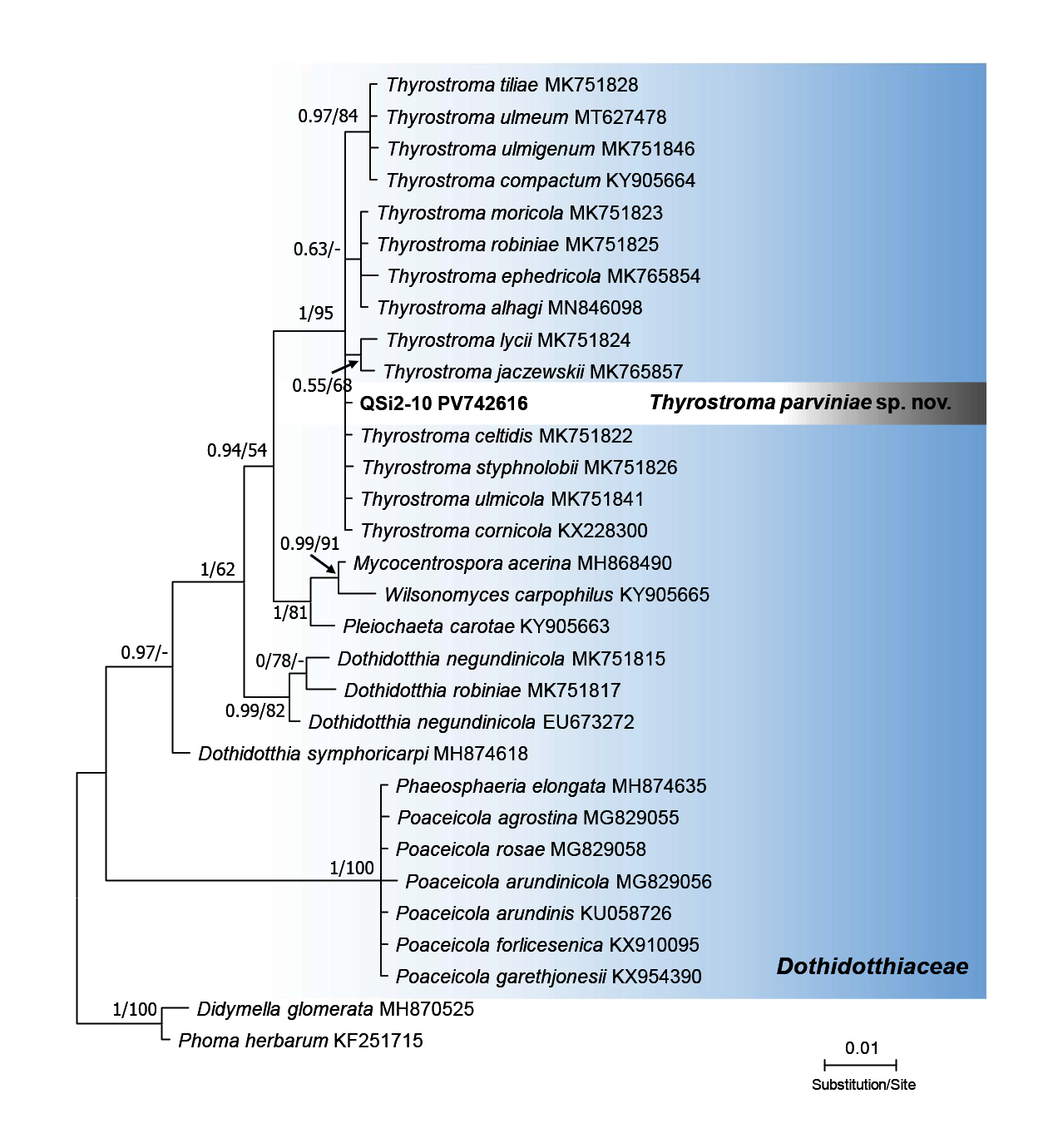

Supplement: S4 Fig — The Bayesian Inference (BI) tree, rooted to outgroup taxa Didymella glomerata (CBS 528.66) and Phoma herbarum (CBS 615.75), illustrates relationships among 28 species in Dothiodotthiaceae based on partial large subunit ribosomal RNA (LSU) gene sequences. Numbers on the nodes indicate Bayesian posterior probability values (BI–PP) followed by Maximum Likelihood bootstrap values (ML–BS). Branches with BI–PP = 1/ML–BS = 100 are considered fully supported. QSi2-10 = ex-type = CBS 154728. The isolate retrieved from infected fig trees in Iran is indicated in bold. Arrows represent the exact position of bootstrap values in the phylogenetic tree. (TIF) [file pone.0341992.s004.tif]

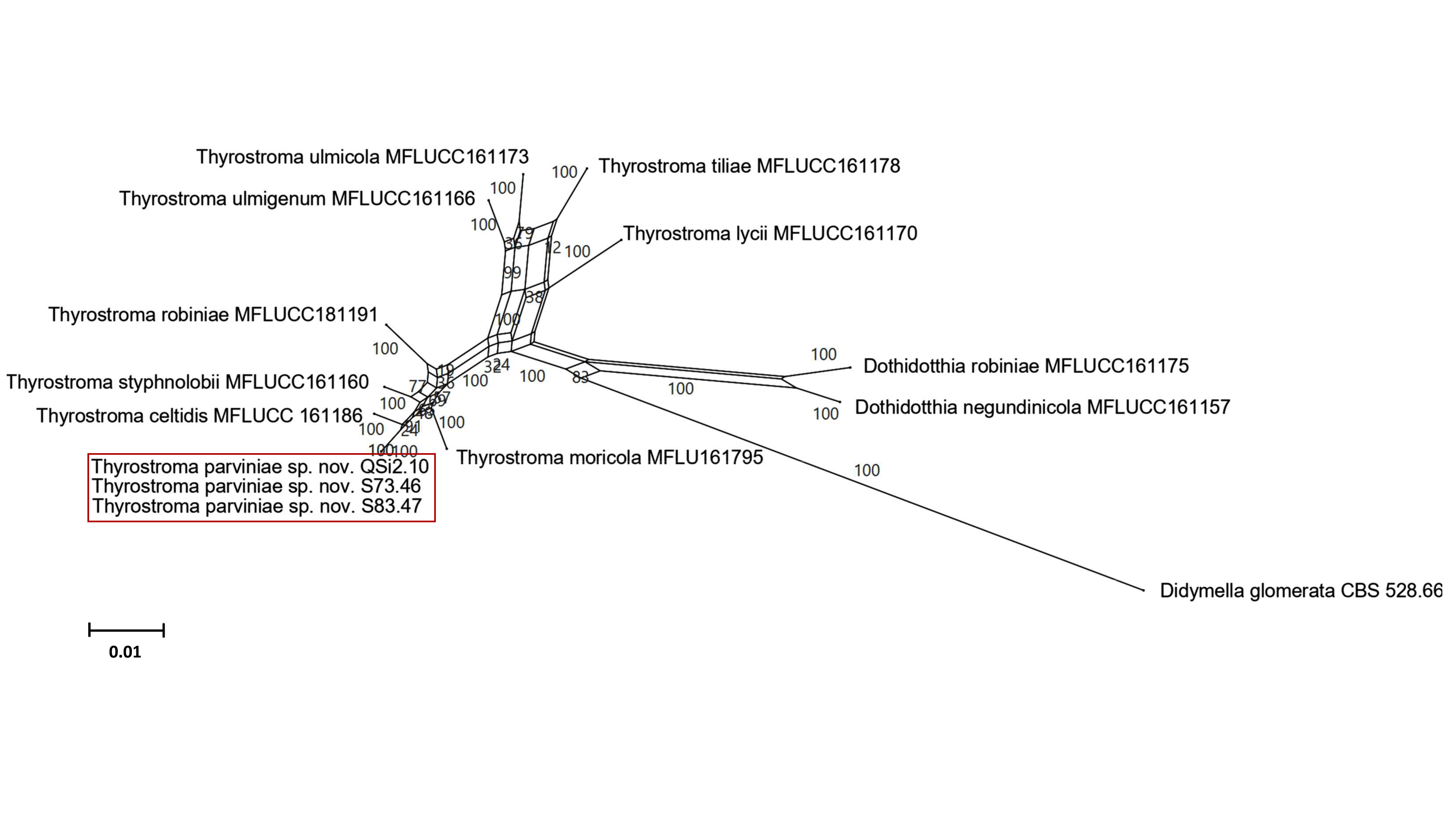

Supplement: S5 Fig — The PHI test was conducted for Thyrostroma parviniae sp. nov. and its closely related species. The isolates of the new species are highlighted in a red box on the graph. (TIF) [file pone.0341992.s005.tif]
