# Supplementary material for: Thyrostroma parviniae sp. nov., causing bud necrosis and branch dieback in fig trees from Iran
Source: PLoS One. 2026 Apr 8;21(4):e0341992. doi: 10.1371/journal.pone.0341992 (PMC13061225; doi:10.1371/journal.pone.0341992)
Supplement: S1 Table — (DOCX) [file pone.0341992.s006.docx]

**S1 Table.** Documented host plants of *Thyrostroma* species.

| **Species** | **Host Plant** | **Family** | **References** |
| --- | --- | --- | --- |
| *Thyrostroma celtidis* | *Celtis occidentalis* L. | *Cannabaceae* | Senwanna et al., 2019 [19] |
| *T. cornicola* | *Amygdalus scoparia* Spach | *Rosaceae* | Mirabdollahi Shamsi et al., 2019  [25] |
|  | *Cornus officinalis* Torr. ex Dur. | *Cornaceae* | Crous et al., 2016 [24] |
| *T. ephedricola* | *Ephedra equisetina* Bunge | *Ephedraceae* | Pem et al., 2019 [23] |
| *T. jaczewskii* | *Elaeagnus angustifolia* L. | *Elaeagnaceae* | Pem et al., 2019 [23] |
| *T. lycii* | *Lycium barbarum* L. | *Solanaceae* | Senwanna et al., 2019 [19] |
| *T. moricola* | *Morus alba* L. | *Moraceae* | Senwanna et al., 2019 [19] |
| *T. robiniae* | *Robinia pseudoacacia* L. | *Fabaceae* | Senwanna et al., 2019 [19] |
| *T. styphnolobii* | *Styphnolobium japonicum* (L.) Schott | *Fabaceae* | Senwanna et al., 2019 [19] |
| *T. tiliae* | *Tilia cordata* Mill. | *Malvaceae* | Senwanna et al., 2019 [19] |
| *T. ulmicola* | *Ulmus pumila* L. | *Ulmaceae* | Senwanna et al., 2019 [19] |
| *T. ulmigenum* | *Ulmus pumila* L. | *Ulmaceae* | Senwanna et al., 2019 [19] |
| *Thyrostroma* sp. | *Vitis vinifera* L. | *Vitaceae* | Travadon et al., 2022 [40] |
