# Supplementary material for: Thyrostroma parviniae sp. nov., causing bud necrosis and branch dieback in fig trees from Iran
Source: PLoS One. 2026 Apr 8;21(4):e0341992. doi: 10.1371/journal.pone.0341992 (PMC13061225; doi:10.1371/journal.pone.0341992)
Supplement: S2 Table — (DOCX) [file pone.0341992.s007.docx]

**S2 Table.** Information of isolates included in the phylogenetic analysis in this study.

| **Species** | **Isolate ^a^** | **Host** | **Host family** | **Location** |  | **GenBank accession no. ^b^** | | | |
| --- | --- | --- | --- | --- | --- | --- | --- | --- | --- |
|  |  |  |  |  |  | **LSU** | **ITS** | ***tef1*** | ***tub2*** |
| *Didymella glomerata* ^c^ | CBS 528.66 | *Chrysanthemum* sp. | *Asteraceae* | Netherlands |  | MH870525 | MW810273 | MW735669 | MW815128 |
| *Dothidotthia negundinicola* | MFLUCC 16-1157 | *Acer negundo* | *Sapindaceae* | Russia |  | MK751815 | MK751725 | MK908015 | MK933784 |
| *Dothidotthia negundinis* | CPC 12928 | *Fendlera rupicola* | *Hydrangeaceae* | USA |  | EU673272 | MK442598 | - | - |
| *Dothidotthia robiniae* | MFLUCC 16-1175 | *Robinia pseudoacacia* | *Fabaceae* | Russia |  | MK751817 | MK751727 | MK908017 | MK933786 |
| *Dothidotthia symphoricarpi* | CBS 119687 | *-* | *-* | USA |  | MH874618 | MH863064 | - | - |
| *Mycocentrospora acerina* | CBS 148.52 | *-* | *-* | USA |  | MH868490 | MH856968 | - | - |
| *Phaeosphaeria elongata* | CBS 120250 | *-* | *-* | Japan |  | MH874635 | MH863080 | GU456261 | - |
| *Phoma herbarum* ^c^ | CBS 615.75 | *Rosa multiflora* | *Rosaceae* | Netherlands |  | KF251715 | KF251212 | - | FJ427133 |
| *Pleiochaeta carotae* | CPC 27452 | *Daucus carota* | *Apiaceae* | South Africa |  | KY905663 | KY905669 | - | - |
| *Poaceicola agrostina* | MFLU 18-0113 | *Agrostis stolonifera* | *Poaceae* | Italy |  | MG829055 | MG828945 | MG829227 | - |
| *Poaceicola arundinicola* | MFLU 16-0225 | *Arundo plinii* | *Poaceae* | Italy |  | MG829056 | MG828946 | MG829228 | - |
| *Poaceicola arundinis* | MFLUCC 15-0702 | *Arundo plinii* | *Poaceae* | Italy |  | KU058726 | KU058716 | MG520921 | - |
| *Poaceicola forlicesenica* | MFLUCC 15-0470 | *Dactylis glomerata* | *Poaceae* | Italy |  | KX910095 | KX926422 | MG520922 | - |
| *Poaceicola garethjonesii* | MFLUCC 15-0469 | *Dactylis glomerata* | *Poaceae* | Italy |  | KX954390 | KX926425 | MG520923 | - |
| *Poaceicola italica* | MFLUCC 13-0267 | *Arundo plinii* | *Poaceae* | Italy |  | KX891169 | KX926421 | MG520924 | - |
| *Poaceicola rosae* | MFLU 18-0114 | *Rosa canina* | *Rosaceae* | Italy |  | MG829058 | MG828948 | MG829230 | - |
| *Thyrostroma alhagi* | MFLUCC 17-1949 | *Alhagi kirghisorum* | *Fabaceae* | Uzbekistan |  | MN846098 | MN846099 | - | - |
| *Thyrostroma celtidis* | MFLUCC 16-1186 | *Celtis occidentalis* | *Cannabaceae* | Russia |  | MK751822 | MK751732 | MK908022 | MK933791 |
| *Thyrostroma compactum* | CBS 335.37 | *Ulmus pumila* | *Ulmaceae* | - |  | KY905664 | KY905670 | - | - |
| *Thyrostroma cornicola* | CPC 25427 | *Cornus officinalis* | *Cornaceae* | South Korea |  | KX228300 | KX228248 | - | - |
| *Thyrostroma ephedricola* | MFLUCC 18-1125 | *Ephedra equisetina* | *Ephedraceae* | Uzbekistan |  | MK765854 | MK765855 | - | - |
| *Thyrostroma jaczewskii* | MFLUCC 18-0787 | *Elaeagnus angustifolia* | *Elaeagnaceae* | Russia |  | MK765857 | MK765856 | - | - |
| *Thyrostroma lycii* | MFLUCC 16-1170 | *Lycium barbarum* | *Solanaceae* | Russia |  | MK751824 | MK751734 | MK908024 | MK933793 |
| *Thyrostroma moricola* | MFLU 16-1795 | *Morus alba* | *Moraceae* | Russia |  | MK751823 | MK751733 | MK908023 | MK933792 |
| *Thyrostroma robiniae* | MFLUCC 18-1191 | *Robinia pseudoacacia* | *Fabaceae* | Russia |  | MK751825 | MK751735 | MK908025 | MK933794 |
| ***Thyrostroma parvina* sp. nov.** | **CBS 154728 ^T^**  **(QSi2-10)** | ***Ficus carica*** | ***Moraceae*** | **Iran** |  | **PV742616** | **PV742613** | **PV750644** | **PV763142** |
| ***Thyrostroma parvina* sp. nov.** | **CBS 154727**  **(S83-47)** | ***Ficus carica*** | ***Moraceae*** | **Iran** |  | **-** | **PV742614** | **PV750645** | **PV763143** |
| ***Thyrostroma parvina* sp. nov.** | **S73-46** | ***Ficus carica*** | ***Moraceae*** | **Iran** |  | **-** | **PV742615** | **PV750646** | **PV763144** |
| *Thyrostroma styphnolobii* | MFLUCC 16-1160 | *Styphonolobium japonicum* | *Fabaceae* | Russia |  | MK751826 | MK751736 | MK908026 | MK933795 |
| *Thyrostroma tiliae* | MFLUCC 16-1178 | *Tilia cordata* | *Malvaceae* | Russia |  | MK751828 | MK751738 | MK908028 | MK933797 |
| *Thyrostroma ulmeum* | MFLU 17-2538 | *hybrid elm Ulmus minor × Ulmus pumila* | *Ulmaceae* | Ukraine |  | MT627478 | MT627476 | MT614250 | - |
| *Thyrostroma ulmicola* | MFLUCC 16-1173 | *Ulmus pumila* | *Ulmaceae* | Russia |  | MK751841 | MK751751 | MK908041 | MK933809 |
| *Thyrostroma ulmigenum* | MFLUCC 16-1166 | *Ulmus pumila* | *Ulmaceae* | Russia |  | MK751846 | MK751756 | MK908046 | MK933814 |
| *Wilsonomyces carpophilus* | CBS 159.51 | *-* | *-* | Italy |  | KY905665 | KY905671 | KY905683 | - |

Note: GenBank accession numbers in bold were newly generated in this study. ^T.^ type isolate.

^a.^ CBS: Westerdijk Fungal Biodiversity Institute, Utrecht, The Netherlands; CPC: Culture collection of Pedro Crous, the Netherlands; MFLU: Mae Fah Luang University Herbarium, Chiang Rai, Thailand; MFLUCC: Mae Fah Luang University Culture Collection, Thailand.

^b.^  ITS: internal transcribed spacers 1 and 2 and 5.8S rRNA gene of rDNA; LSU: partial 28S large subunit RNA gene; *tef1*: partial translation elongation factor 1–alpha gene; *tub2*: partial β-tubulin gene.

^c.^ Outgroup
