# Supplementary material for: Thyrostroma parviniae sp. nov., causing bud necrosis and branch dieback in fig trees from Iran
Source: PLoS One. 2026 Apr 8;21(4):e0341992. doi: 10.1371/journal.pone.0341992 (PMC13061225; doi:10.1371/journal.pone.0341992)
Supplement: S3 Table — (DOCX) [file pone.0341992.s008.docx]

**S3 Table.** Nucleotide differences across LSU, ITS, *tef1*, and *tub2* loci illustrating interspecific variation between *Thyrostroma parviniae* sp. nov. and related species.

| Region | Alignment length (bp) | Differences with *T. celtidis* (bp) | Differences with *T. lycii* (bp) | Differences with *T. moricola* (bp) | Differences with *T. robiniae* (bp) | Differences with *T. styphnolobii* (bp) | Differences with *T. tiliae* (bp) | Differences with *T. ulmeum* (bp) | Differences with *T. ulmicola* (bp) | Differences with *T. ulmigenum* (bp) |
| --- | --- | --- | --- | --- | --- | --- | --- | --- | --- | --- |
| LSU | 709 | 0 | 1 | 1 | 1 | 0 | 2 | 2 | 0 | 2 |
| ITS | 470 | 3 | 14 | 3 | 1 | 1 | 18 | 19 | 13 | 15 |
| *tef1* | 607 | 4 | 17 | 8 | 9 | 7 | 17 | 23 | 20 | 18 |
| *tub2* | 295 | 6 | 32 | 11 | 16 | 10 | 34 | NA* | 30 | 27 |
| Total | 2081 | 13 | 64 | 23 | 27 | 18 | 71 | NA | 63 | 62 |

LSU: partial 28S large subunit RNA gene; ITS: internal transcribed spacers 1 and 2 and 5.8S rRNA gene of rDNA; *tef1*: partial translation elongation factor 1–alpha gene; *tub2*: partial β-tubulin gene. *Data not available
