# Supplementary material for: Thyrostroma parviniae sp. nov., causing bud necrosis and branch dieback in fig trees from Iran
Source: PLoS One. 2026 Apr 8;21(4):e0341992. doi: 10.1371/journal.pone.0341992 (PMC13061225; doi:10.1371/journal.pone.0341992)
Supplement: S4 Table — (DOCX) [file pone.0341992.s009.docx]

**S4 Table.** Morphometric measurements of conidia, conidiogenous cells and conidiophore from *Thyrostroma parviniae* sp. nov. isolates collected from infected fig trees in Fars Province, Iran.

| **Isolate** | **Conidia** | | | | | | |  | **Conidiogenous cell** | | | | | | |
| --- | --- | --- | --- | --- | --- | --- | --- | --- | --- | --- | --- | --- | --- | --- | --- |
|  | **Length** | | |  | **Width** | | |  | **Length** | | |  | **Width** | | |
|  | Minimum | Maximum | Average |  | Minimum | Maximum | Average |  | Minimum | Maximum | Average |  | Minimum | Maximum | Average |
| **S83-47** | 32.9 | 66.6 | 47.9 ± 7.8^*^ |  | 13.2 | 32.7 | 23.2 ± 4.7 |  | 7.6 | 23.9 | 14.9 ± 4.1 |  | 6.8 | 13.6 | 9.5 ± 1.9 |
| **S73-46** | 25.4 | 49.8 | 36.6 ± 5.6 |  | 14.8 | 26.9 | 19.1 ± 2.8 |  | 5.9 | 15.6 | 10.9 ± 2.2 |  | 6.0 | 12.6 | 8.5 ± 1.5 |
| **QSi2-10** | 26.4 | 58.5 | 46.1 ± 8.3 |  | 16.2 | 30.8 | 24.7 ± 3.8 |  | 7.7 | 19.8 | 13.2 ± 2.8 |  | 7.6 | 12.6 | 9.9 ± 1.5 |
| **QSi2-11** | 23.3 | 65.4 | 47.7 ± 10.3 |  | 22.1 | 33.4 | 27.3 ± 3.0 |  | 8.2 | 18.2 | 11.3 ± 2.6 |  | 7.5 | 13.3 | 10.1 ± 2.0 |
| **QSi2-13** | 22.8 | 77.9 | 41.5 ± 9.7 |  | 17.0 | 34.3 | 25.7 ± 3.9 |  | 6.0 | 18.6 | 11.7 ± 2.9 |  | 7.8 | 15.5 | 10.9 ± 2.4 |
| **Mean** | **21.2 ± 4.2** | **63.6 ± 10.4** | **44.0 ± 8.3** |  | **16.7 ± 3.4** | **31.6 ± 2.9** | **24.0 ± 3.6** |  | **7.1 ± 1.1** | **19.2 ± 3.0** | **12.4 ± 2.9** |  | **7.1 ± 0.7** | **13.5 ± 1.2** | **9.8 ± 1.9** |

**S4 Table.** Continue.

| **Isolates** | **Conidiophore** | | | | | | |
| --- | --- | --- | --- | --- | --- | --- | --- |
|  | **Length** | | |  | **Width** | | |
|  | Minimum | Maximum | Average |  | Minimum | Maximum | Average |
| **S83-47** | 30.1 | 65.5 | 44.3 ± 9.5^*^ |  | 4.9 | 9.7 | 7.3 ± 1.2 |
| **S73-46** | 40.6 | 72.5 | 51.9 ± 8.5 |  | 4.2 | 9.9 | 7.3 ± 1.5 |
| **QSi2-10** | 42.6 | 73.7 | 54.5 ± 8.9 |  | 4.3 | 7.2 | 5.9 ± 0.8 |
| **QSi2-11** | 41.3 | 69.2 | 55.5 ± 7.5 |  | 4.5 | 7.8 | 6.5 ± 1.0 |
| **QSi2-13** | 40.9 | 66.3 | 57.5 ± 6.7 |  | 5.1 | 8.5 | 6.9 ± 1.1 |
| **Mean** | **39.1 ± 5.1** | **69.4 ± 3.6** | **52.7 ± 8.2** |  | **4.6 ± 0.39** | **8.6 ± 1.2** | **6.8 ± 1.1** |

**^*^**Mean ± standard deviation (µm)
