# Supplementary material for: Thyrostroma parviniae sp. nov., causing bud necrosis and branch dieback in fig trees from Iran
Source: PLoS One. 2026 Apr 8;21(4):e0341992. doi: 10.1371/journal.pone.0341992 (PMC13061225; doi:10.1371/journal.pone.0341992)
Supplement: S5 Table — (DOCX) [file pone.0341992.s010.docx]

**S5 Table.**  One-way Analysis of variance (ANOVA) results of four pathogenicity traits on one-year-old branches of fig trees inoculated with Thyrostroma parviniae sp. nov.

| **S.O.V.** | **df** | **Lesion length** | | |  | **Lesion width** | | |  | **Upward internal lesion progression** | | |  | **Downward internal lesion progression** | | |
| --- | --- | --- | --- | --- | --- | --- | --- | --- | --- | --- | --- | --- | --- | --- | --- | --- |
|  |  | MS | F-value | *P*-value |  | MS | F-value | *P*-value |  | MS | F-value | *P*-value |  | MS | F-value | *P*-value |
| **Isolate** | 30 | 17.61 | 29.64 | <0.0001 |  | 11.68 | 37.68 | <0.0001 |  | 159.66 | 76.3 | <0.0001 |  | 93.55 | 51.15 | <0.0001 |
| **Error** | 62 | 0.59 |  |  |  | 0.31 |  |  |  | 2.09 |  |  |  | 1.83 |  |  |
| **CV (%)** |  |  | 4.70 |  |  |  | 5.81 |  |  |  | 7.55 |  |  |  | 9.05 |  |

CV: Coefficient of variation; df: Degrees of freedom; MS: Mean Square; S.O.V.: Source of variation.
